# New fluorogenic triacylglycerols as sensors for dynamic measurement of lipid oxidation

**DOI:** 10.1007/s00216-024-05642-w

**Published:** 2024-11-21

**Authors:** Maria Handke, Frank Beierlein, Petra Imhof, Matthias Schiedel, Simon Hammann

**Affiliations:** 1 Department of Chemistry and Pharmacy, Friedrich-Alexander-Universität Erlangen-Nürnberg, Nikolaus-Fiebiger-Straße 10, Erlangen, 91058 Germany; 2 Institute of Medicinal and Pharmaceutical Chemistry, Technische Universität Braunschweig, Beethovenstraße 55, Braunschweig, 38106 Germany; 3 Computer Chemistry Center, Friedrich-Alexander-Universität Erlangen-Nürnberg, Nägelsbachstraße 25, Erlangen, 91052 Germany; 4 Zentrum für Nationales Hochleistungsrechnen Erlangen (NHR@FAU), Martensstraße 1, Erlangen, 91058 Germany; 5 FAU NeW - Research Center for New Bioactive Compounds, Nikolaus-Fiebiger-Str. 10, Erlangen, 91058 Germany; 6 Department of Food Chemistry and Analytical Chemistry (170a), Institute of Food Chemistry, University of Hohenheim, Garbenstraße 28, Stuttgart, 70599 Germany

**Keywords:** Lipid oxidation, Fluorogenic substrate, Triacylglycerol, Assay, Click chemistry

## Abstract

Lipids are major constituents of food but are also highly relevant substructures of drugs and are increasingly applied for the development of lipid-based drug delivery systems. Lipids are prone to oxidative degradation, thus affecting the quality of food or medicines. Therefore, analytical methods or tools that enable the degree of lipid oxidation to be assessed are of utmost importance to guarantee food and drug safety. Herein, we report the design, synthesis and application of the first-in-class fluorogenic triacylglycerols that enable dynamic monitoring of lipid oxidation via straightforward fluorescence readout. Our fluorogenic triacylglycerols can be used in both aqueous and lipid-based environments. Furthermore, we showed that the sensitivity of our fluorescent tracers towards oxidation could be tuned by incorporating either saturated or unsaturated acyl chains in their triacylglycerol core structure. With this, we provide a first proof of principle for the applicability of fluorescently labelled triacylglycerols as tracers to monitor the dynamics of lipid oxidation, thus paving the way for novel discoveries in the area of lipid analytics.

**Graphical Abstract:**

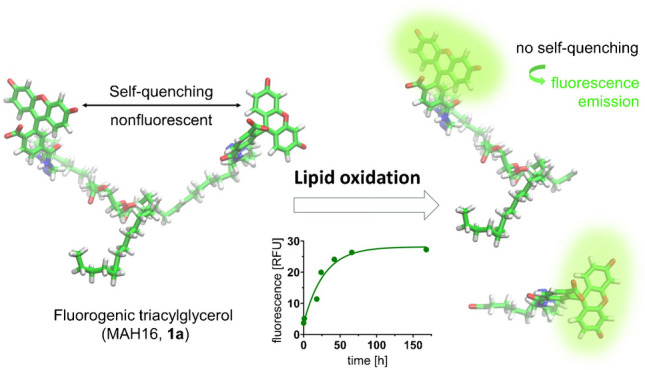

**Supplementary Information:**

The online version contains supplementary material available at 10.1007/s00216-024-05642-w.

## Introduction

Lipids fulfil multifaceted roles in living organisms and are also one of the major constituents of most food we consume. In food, they are important contributors to caloric value, contribute to texture as well as mouthfeel and act as precursors for odour-active compounds. In addition, numerous lipids exhibit specific bioactivities, thus making them a beneficial or even essential part of human diet. Lipids are a highly diverse group of organic compounds including, among others, plant sterols; the fat-soluble vitamins A, E, D and K; as well as saturated and (poly-)unsaturated fatty acids [1]. Beyond food, lipids are also highly relevant substructures of drugs (e.g. daptomycin, latanoprost, calcitriol, mupirocin) and are increasingly applied for the development of lipid-based drug delivery systems (LBDDS) [2], thus highlighting their importance in both drug discovery and drug development.

**Fig. 1:**
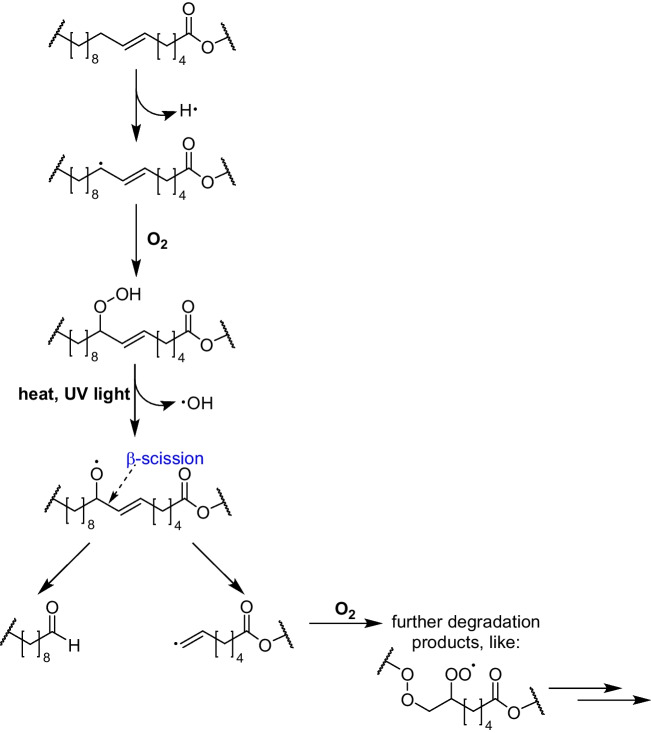
Autoxidation mechanism of unsaturated lipids and possible degradation products. Figure adapted from Schaich [5] and Alireza et al. [6]

Due to the frequently high number of double bonds in their structures, lipids are very susceptible to oxidative degradation, which alters their structures leading to a loss of their beneficial properties and the formation of degradation products with undesired olfactory or bioactive characteristics [3, 4]. In general, the susceptibility of lipids towards oxidations increases with the number of double bonds in their structure as radicals can be mesomerically stabilized in allylic positions. For example, the oxidation rate of mono-unsaturated fatty acids is approximately 100 times higher than that of saturated fatty acids lacking this mesomeric stabilization of the initially formed radical [3]. In the initial phase of lipid oxidation, hydroperoxides are formed, either through radical-based autoxidation, photooxygenation or the action of lipoxygenases [3]. These hydroperoxides are highly reactive species and react to a multitude of secondary or tertiary oxidation products, including odour-active aldehydes formed by alpha and beta scission (Fig. 1) [5].

Accordingly, hydroperoxide concentrations can be low in highly oxidized oils, since the hydroperoxides have already reacted to further degradation products [7]. This makes the determination of the state and dynamics of lipid oxidation by frequently employed wet chemistry techniques (e.g. peroxide value, anisidine value), targeted at specific classes of lipid oxidation products (e.g. peroxides, aldehydes), highly challenging and prone to error [7]. On the other hand, more sophisticated and comprehensive methods such as (hyphenated) mass spectrometry techniques or nuclear magnetic resonance (NMR) spectroscopy require extensive sample preparation and/or expensive instrumentation.

Herein, we aimed for developing fluorogenic lipid analogues as molecular tools to study the dynamics of lipid oxidation via straightforward fluorescence readout. These reporter molecules are triacylglycerols with two of their fatty acid chains conjugated to a fluorescein dye. In the intact tracer molecule, the spatial proximity of the two fluorophores leads to a radiationless resonance energy transfer between these entities, also referred to as Förster resonance energy transfer (FRET), resulting in a self-quenching effect concomitant with low fluorescence emission. If one of the fluorophores is cleaved off from the molecule and leaves the distance for efficient resonance energy transfer (> 10 nm), an increase in fluorescence signal can be detected. This principle has previously led to the application of similar molecules as tracers for lipase activity assays [8]. We now aimed for transferring this concept to detect lipid oxidation, by incorporating unsaturated and thus oxidation-labile fatty acids in our fluorescent tracers. Analogously to native lipids, the unsaturated fatty acid chain(s) of our tracer molecule can be cleaved via beta scission of the initially formed hydroperoxides, resulting in an increase in fluorescence. Our fluorescent tracer molecules are synthesized from easily available and affordable starting materials in a click chemistry–based approach and enabled a time-resolved detection of lipid oxidation in food and other matrices.

## Materials and methods

### Computational methods

For conformational analysis, the fluorogenic tracers **1a** and **1b**, respectively, were each solvated in a truncated octahedral methanol box exceeding the organic molecules by 15 Å on either side [9]. Sodium ions [10] were added to neutralize the di-anionic fluorescein moieties. The di-anionic state of fluorescein was used since this is the predominant form at neutral pH values [11, 12]. For the solute molecules, we used GAFF2 [13, 14] parameters with RESP [15, 16] charges based on calculations with Gaussian 16 [17] (HF/6-31G*//B3LYP/6-31G*) [18–28] (optimizations and frequency calculations were performed in polarizable continuum model (PCM) water [29, 30]) in agreement with the Amber force fields [15, 31]. After initial geometry optimization (first 5000 steps with restraints (50 kcal mol^−1^ Å^−2^) on the ligands, then 5000 optimization steps without restraints, switch from the steepest descent to conjugate gradients after 500 steps in either case), the solvated systems were heated to 298 K during a 500-ps simulation with weak restraints (10 kcal mol^−1^ Å^−2^) on the ligands in the NVT ensemble. After that, three independent production runs of 1000-ns unrestrained NPT Langevin dynamics with a time step of 2 fs were performed for each simulation system at 298 K and 1 bar (weak pressure coupling, isotropic position scaling, pressure relaxation time 2 ps, collision frequency 2 ps^−1^), each starting with individual initial velocities, using Amber 23 [32]. SHAKE constraints were applied to bonds involving hydrogen [33]. Periodic boundary conditions were used throughout, and the distance cut-off for all nonbonding interactions was set to 10 Å. Long-range electrostatics were described by the particle-mesh Ewald method [34, 35]. For van der Waals interactions beyond those included in the direct sum, a continuum model correction for energy and pressure was used, as implemented in Amber. Coordinates were saved every 100 ps.

From these simulations, the first 100 ns was dismissed as equilibration and only the remaining 900 ns was used for analysis. All properties are reported as average over the three replicas. Error estimates were computed as standard error from the mean of these three replicas. Analyses were performed using cpptraj [36], and meta-analyses and plotting were done with python [37] and matplotlib [38], using Jupyter notebooks [39]. Molecules were visualized with VMD [40]. Transition dipole vectors of the fluorescein moieties were assumed to lie in the plane of the xanthene chromophores as shown in our previous work [41].

### Fluorescence-based assays

#### Fluorescence-based detection of alkaline triacylglycerol hydrolysis

The tracer solutions were prepared by the addition of 10 µL of a 10 mM stock solution of the respective tracer (in methanol) to 1.0 mL of a 5% KOH solution in water/ethanol solution (1:9, *v*/*v*). For our control experiments, we added the tracer solutions to 1.0 mL of a water/ethanol solution (1:9, *v*/*v*) instead. The reaction mixtures were stirred at 80 °C and protected from daylight. After a certain incubation time, samples (1.0 µL) were taken, diluted with 1.0 mL methanol, and fluorescence was recorded. The fluorescence measurements were carried out using a FP-6200 spectrofluorometer from Jasco with an excitation wavelength of 485 nm (bandwidth, 5 nm) and a detection wavelength of 514 nm (bandwidth, 5 nm) with high sensitivity. All measurements were performed in triplicate.

#### Fluorescence-based detection of lipid oxidation

For experiments in an aqueous environment, we referred to the conditions described by Sjövali et al. [49]. Ten microlitres of a 10 mM stock solution of the respective fluorogenic tracer (in methanol) was added to 1.0 mL of an aqueous 70% *t*-butyl-hydroperoxide (TBHP) solution. Additionally, 10 µL of an aqueous 10 µM FeSO_4_ solution and 100 µL of aqueous 0.2% taurocholic acid solution were added. The reaction mixtures were incubated protected from daylight in a closed reaction vessel at 40 °C while stirring. After a certain incubation time, samples (20 µL) were taken and diluted with 1.0 mL methanol, and fluorescence was recorded as described above. All measurements were performed in triplicate.

For experiments in a lipid-based environment, 10 µL of a 10 mM stock solution of the respective fluorogenic tracer (in methanol) was added to 30 mg of glycerol trioleate. The reaction mixtures were incubated in an open reaction vessel at 40 °C while stirring. After a certain incubation time, samples were taken for fluorescence readout. For this, we added 1.0 mL of methanol to the reaction mixture and used 30 µL thereof for fluorescence readout. Another 30 µL of this mixture was used for ^1^H NMR analysis (see below). The remaining reaction mixture was dried under a nitrogen stream to remove the methanol, and further incubated applying the aforementioned conditions. The 30 µL of the mixture that was taken for fluorescence readout was further diluted with 1.0 mL methanol, and fluorescence was recorded as described above. All measurements were performed in triplicate.

### ^1^H NMR–based detection of lipid oxidation

The 30 µL samples that were taken for ^1^H NMR analyses from the oxidation experiments in a lipid-based environment (see above) were dried under a nitrogen stream to remove the methanol. Subsequently, the residue was dissolved in 500 µL of CDCl_3_ for ^1^H NMR measurements.

## Results and discussion

### Design of the fluorogenic triacylglycerols

**Fig. 2:**
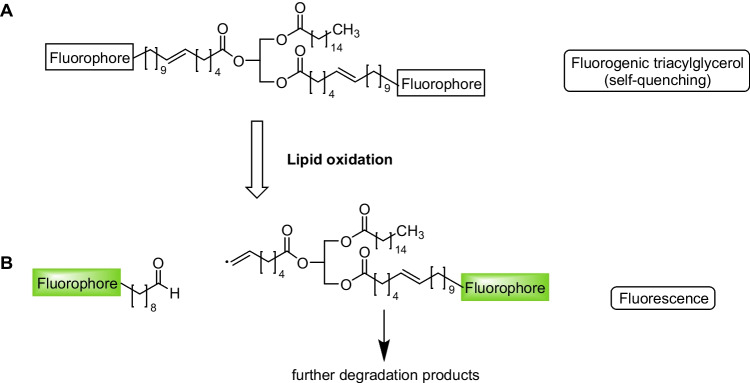
Design of fluorogenic triacylglycerols. **A** In the intact tracer molecule, radiationless resonance energy transfer between the two fluorophores results in a self-quenching effect. **B** During lipid oxidation, the fluorophore(s) can be cleaved off from the triacylglycerol core structure, thus leading to a termination of self-quenching and an increase in fluorescence

For the design of our triacylglycerol-based fluorogenic substrates to study lipid oxidation, we aimed for installing two fluorophore-labelled unsaturated fatty acid chains and one nonfluorescent saturated fatty acid chain at the three hydroxyl groups of glycerol (Fig. 2).

**Fig. 3:**
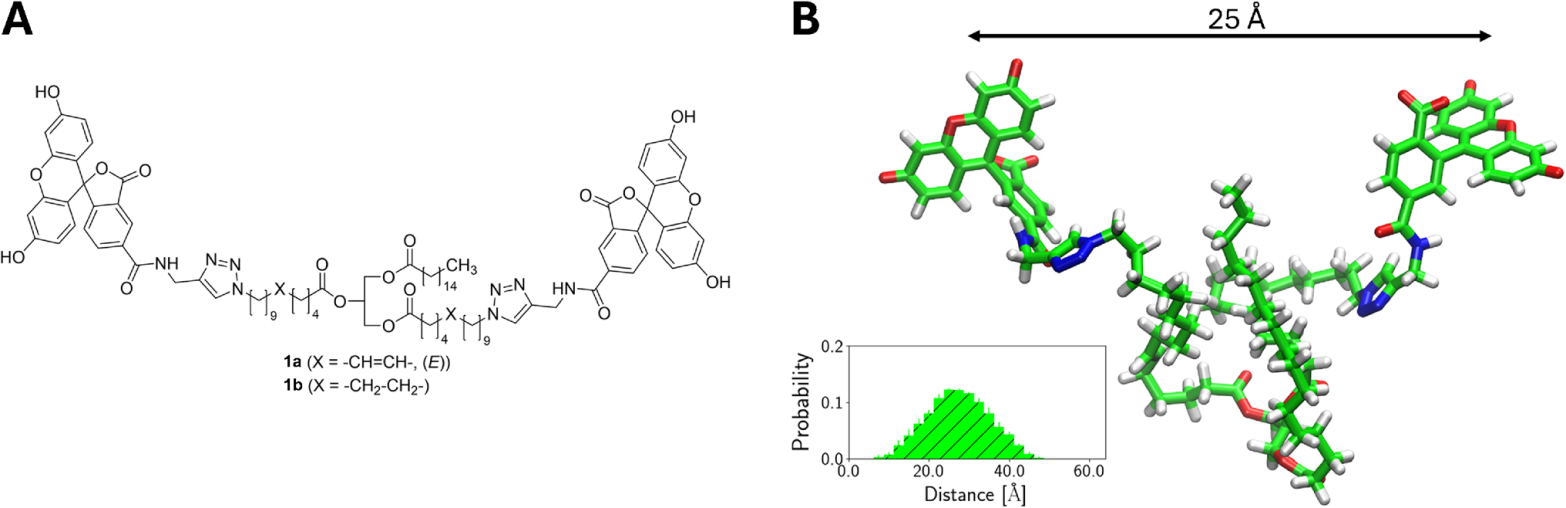
Chemical structures of the envisaged fluorogenic triacylglycerols **1a** and **1b**. **A** Two-dimensional chemical structures of **1a** as a molecular tracer to study lipid oxidation and **1b** as a control compound that should be less prone to lipid oxidation due to its saturated acyl chains. **B** Medoid conformation of **1a** as predicted by MD simulations. Inset shows the predicted distribution of distances between the two fluorophore labels of **1a**. The medoid structure of **1b** can be found in Supplementary Fig. 2

As our fluorogenic substrates should resemble the structural and chemical properties of natural triacylglycerols as closely as possible, we chose, in analogy to the ubiquitous fatty acid residues with a 16-carbon chain (i.e. palmitic acid or palmitoleic acid), this chain length for the acyl residues of our model substrate. To enable straightforward fluorescence labelling at a final stage, we wanted to apply the Cu(I)-catalysed azide-alkyne cycloaddition (CuAAC), also referred to as click reaction, thereby leading to triazole-based linker units between the ω-carbon atoms of the unsaturated fatty acid chains and the fluorescein label. These triazole-based linker units are chemically stable and not susceptible to hydrolytic or oxidative cleavage, which is ideal for our purpose. As a fluorophore for fluorescence labelling, we used fluorescein, due to its affordable commercial availability and its prior application for the development of similar triacylglycerol-based fluorogenic substrates for lipases [8]. Based on the aforementioned information, we defined MAH16 (**1a**; Fig. 3A) as a target structure for our fluorogenic substrate to study lipid oxidation. In order to validate that the distance between the two fluorophores of the intact tracer molecule **1a** is below 100 Å (= 10 nm), thus enabling fluorescence self-quenching via FRET, we predicted the 3D structure by molecular dynamics (MD) simulations and conformational analyses. Our predictions indicated that **1a** exhibits rich conformational dynamics as manifested by the broad range of distances (7.5–47.5 Å) between the two fluorophores with a major population around 25 Å, which should enable highly efficient radiationless resonance energy transfer between these entities (Fig. 3B and Supplementary Fig. 1A–C) [50]. MAH12 (**1b**; Fig. 3A), which exclusively features saturated acyl chains, was designed as a reference compound with very similar hydrolytic stability but much weaker susceptibility to lipid oxidation, as compared to **1a**. In order to investigate if **1b** also shows similar FRET-relevant structural features as **1a**, especially regarding the distance between its two fluorophores, we also performed MD simulations and conformational analyses for this compound. Despite its higher degree of rotational freedom, due to its fully saturated acyl chains, we predicted for **1b** highly similar structural properties as compared to **1a**, including the distribution of distances between the fluorophore labels (Supplementary Figs. 1 and 2), radii of gyration (Supplementary Fig. 3) and relative fluorophore orientations (Supplementary Figs. 4–6). Thus, these two molecules should differ in their susceptibility towards lipid oxidation but not in their FRET-relevant structural features.

### Chemical synthesis

**Scheme 1:**
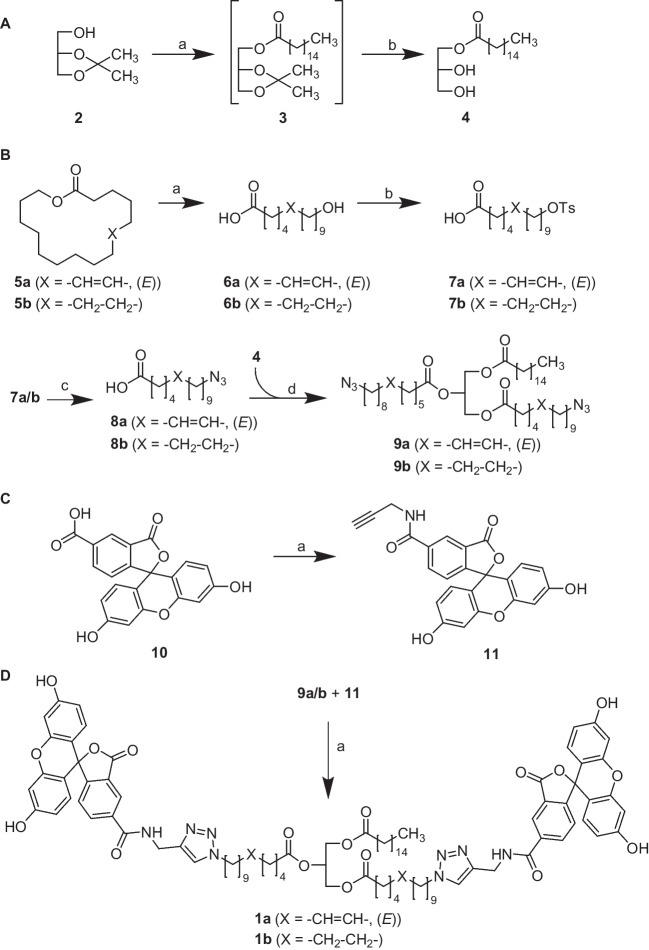
Synthesis of fluorogenic lipid analogues. Reagents and conditions. **A** Synthesis of the monoacylglycerol **4**: (a) palmitic acid, DMAP, DCC, DMF, rt, overnight; (b) 80% acetic acid, rt, overnight, yield (over two steps) 73%. **B** Synthesis of clickable triacylglycerol **9a** and **9b**: (a) KOH, EtOH, 80 °C, overnight, yield 93–95%; (b) tosyl chloride, pyridine, THF, rt, 3 h, yield 35–81%; (c) NaN_3_, DMF, 80 °C, overnight, yield 44–97%; (d) **3**, DMAP, DCC, rt, overnight, yield 25–46%. **C** Synthesis of 5-FAM-alkyne (**11**): (a) HOBT, DIC, TEA, DMF, rt, overnight, yield:41%. **D** Synthesis of the fluorogenic triacylglycerols **1a** and **1b**: (a) sodium ascorbate, CuSO_4_·5H_2_O, TBTA, EtOH/DMF (4:1 (*v*/*v*)), 41 °C, 3 h, yield 53–58%

To enable a versatile approach that allows a final-stage fluorescence labelling of the triacylglycerol core structure via CuAAC, we first established a synthesis protocol for azido-functionalized fatty acids and triacylglycerols, respectively (Scheme 1A, B). In brief, the monoacylglycerol (**4**) bearing a single palmitoyl group in position 1 was synthesized by a Steglich esterification with dl-1,2-isopropylideneglycerol (**2**, also referred to as solketal) and palmitic acid to obtain the intermediate **3** [42, 43]. Then, the acetonide moiety of **3** was hydrolyzed to release the monoacylglycerol **4** with its vicinal diol substructure. The azido-functionalized fatty acids (**8a** and **8b**) that were used for acylating the two hydroxyl groups of **4** were synthesized starting from a commercially available ω-6-hexadecenlactone (**5a**) and hexadecanolactone (**5b**), respectively. Hydrolysis of the lactones (**5a** and **5b**) yielded (*E*)-16-hydroxyhexadec-6-enoic acid (**6a**) and 16-hydroxyhexadecanoic acid (**6b**) [45], respectively, which were subsequently tosylated [46] to obtain **7a** and **7b**. S_N_2 reaction of **7a** and **7b** with sodium azide gave the azido-functionalized fatty acids **8a** and **8b**. A Steglich esterification [43] with **8a**, **8b** and **4** resulted in the triacylglycerols **9a** and **9b** featuring one palmitoyl and two ω-azido-functionalized acyl residues. The alkynylated fluorescein analogue (**11**, 5-FAM-alkyne) was synthesized by an amide coupling of 5-carboxyfluorescein (**10**, 5-FAM) with propargyl amine, according to a previously reported procedure (Scheme 1C) [48]. In the final step of the synthesis, the obtained azido-functionalized triacylglycerols **9a** and **9b** were conjugated with an alkynylated fluorescein analogue (**11**) via CuAAC [51–53], to obtain the final fluorogenic triacylglycerols **1a** and **1b** (Scheme 1D). Due to the poor solubility of **9a** and **9b** in aqueous solvent mixtures, we used ethanol as a solvent for the click reaction and performed the reaction at an elevated temperature (41 °C).

### Application of fluorogenic triacylglycerols

**Fig. 4:**
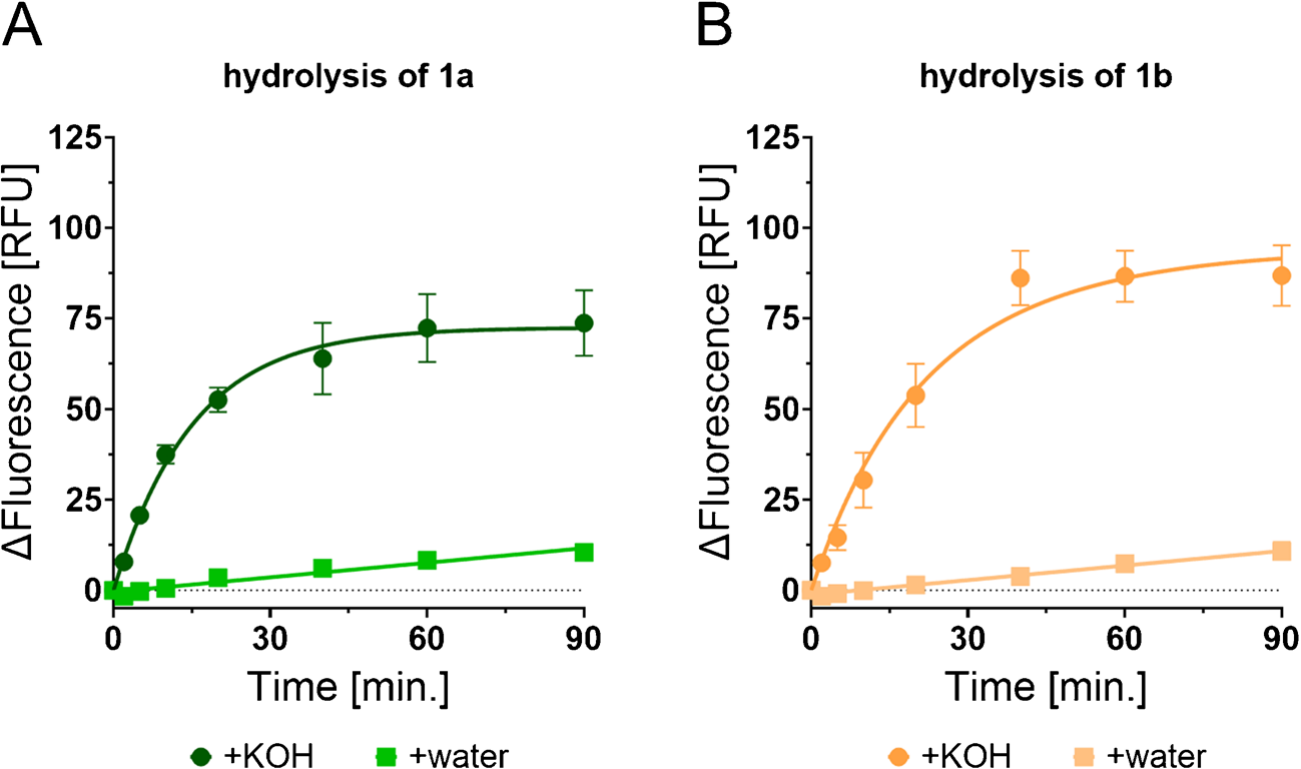
Time course of the hydrolysis of fluorogenic tracers **1a** and **1b** in the presence or absence of KOH (0.88 M). **A** Fluorescence signal (mean ± SD) over time for compound **1a**. **B** Fluorescence signal (mean ± SD) over time for compound **1b**. All measurements were performed in triplicate (*n* = 3). Values were normalized to their fluorescence signal at *t* = 0 min (see Supplementary Tables 1–4 for non-normalized raw data)

Next, the synthesized fluorogenic triacylglycerols were tested regarding their applicability as molecular tools to study the dynamics of lipid spoilage. Before applying our fluorogenic triacylglycerols as molecular tracers for lipid oxidation, we first aimed for providing a proof of principle that a cleavage of the triacylglycerol core structure of our fluorogenic tracers, concomitant with the release of at least one of the two attached fluorophores, results in a termination of self-quenching and a significant increase in fluorescence. To this end, we subjected our fluorogenic substrates to the relatively harsh conditions of alkaline ester hydrolysis (KOH (5%) in H_2_O/EtOH (1:9 (*v*/*v*)), 80 °C). For both tracers **1a** and **1b**, we observed a significant increase in fluorescence over time with a typical hyperbolic curve profile (Fig. 4A, B and Supplementary Tables 1–4). In both cases, a maximum signal was reached after 40 to 60 min. Our control experiments in the absence of KOH indicated that despite the presence of water and elevated temperatures (H_2_O/EtOH (1:9 (*v*/*v*)), 80 °C), our fluorogenic tracers are relatively stable towards hydrolysis.

After this initial proof of principle that a cleavage of the triacylglycerol core structure of our fluorogenic tracers results in a significant increase in fluorescence, we aimed for studying the applicability of our fluorogenic tracer molecules to monitor lipid oxidation. Therefore, we subjected our tracers to two different model systems.

**Fig. 5:**
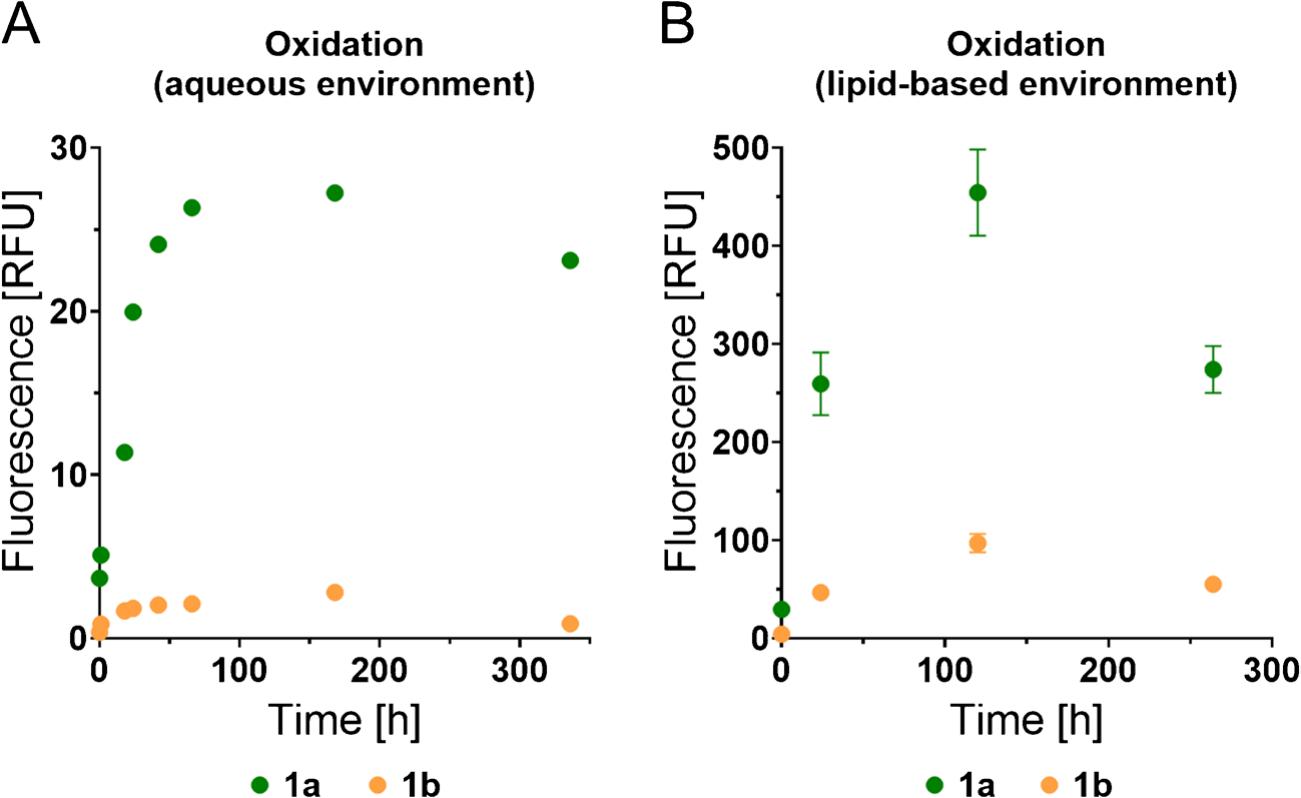
Application of fluorogenic tracers **1a** and **1b** as molecular tools to detect lipid oxidation. **A** Time course of lipid oxidation as detected by means of our fluorogenic tracers **1a** and **1b** in an aqueous environment (mean ± SD, triplicate measurements, water, TBHP, FeSO_4_, taurocholic acid, light-protected at 40 °C). See Supplementary Tables 5 and 6 for raw data. **B** Time course of lipid oxidation as detected by means of our fluorogenic tracers **1a** and **1b** in a lipid-based environment using ambient oxygen as an oxidant (mean ± SD, triplicate measurements, *n* = 3, glycerol trioleate, open reaction vessel, 40 °C). See Supplementary Tables 7 and 8 for raw data

In an aqueous model system, for which we referred to the conditions reported by Sjövali et al. [49], we used TBHP in the presence of FeSO_4_ for creating an oxidative environment. In addition, taurocholic acid was used as a detergent and the samples were incubated in a light-protected closed reaction vessel at 40 °C over time. For our tracer **1a** with its two fluorescently labelled unsaturated fatty acid chains, we detected a robust and time-dependent increase of the fluorescence signal, indicating an oxidative chain cleavage and the release of at least one of the two fluorophores (Fig. 5A and Supplementary Table 5). Until the seventh day of the measurement, the detected values lie, as expected, very nicely on a hyperbolic curve. At a later time point (i.e. 336 h), we observed a decrease in fluorescence, which might at least in part be attributed to the limited stability of fluorescein towards oxidation [54]. For our control compound **1b**, we detected a much weaker increase in fluorescence over time (Fig. 5A and Supplementary Table 6), which can be rationalized by its much lower susceptibility towards lipid oxidation as a consequence of its fully saturated fatty acid chains. Consistent with the results for **1a**, we also observed a certain decrease in fluorescence after 336 h of incubation for **1b**.

Finally, we aimed for investigating our fluorogenic substrates as tracers for lipid oxidation in a lipid-based environment. To this end, we incubated our fluorogenic substrates in glycerol trioleate in an open reaction vessel using ambient oxygen as an oxidant, thus simulating a genuine real-world application. The samples were incubated at 40 °C. Highly consistent with the data from our aqueous model, we also observed a strong increase in fluorescence for our tracer **1a** with its two fluorescently labelled unsaturated fatty acid chains until 120 h of incubation (Fig. 5B and Supplementary Table 7). For the control compound **1b**, derived from a fully saturated triacylglycerol, a significantly lower conversion was detected (Fig. 5B and Supplementary Table 8). For the longest incubation time (i.e. 264 h), we detected a decrease in fluorescence for both **1a** and **1b**, which is again in good agreement with the data from the aqueous setup.

**Fig. 6:**
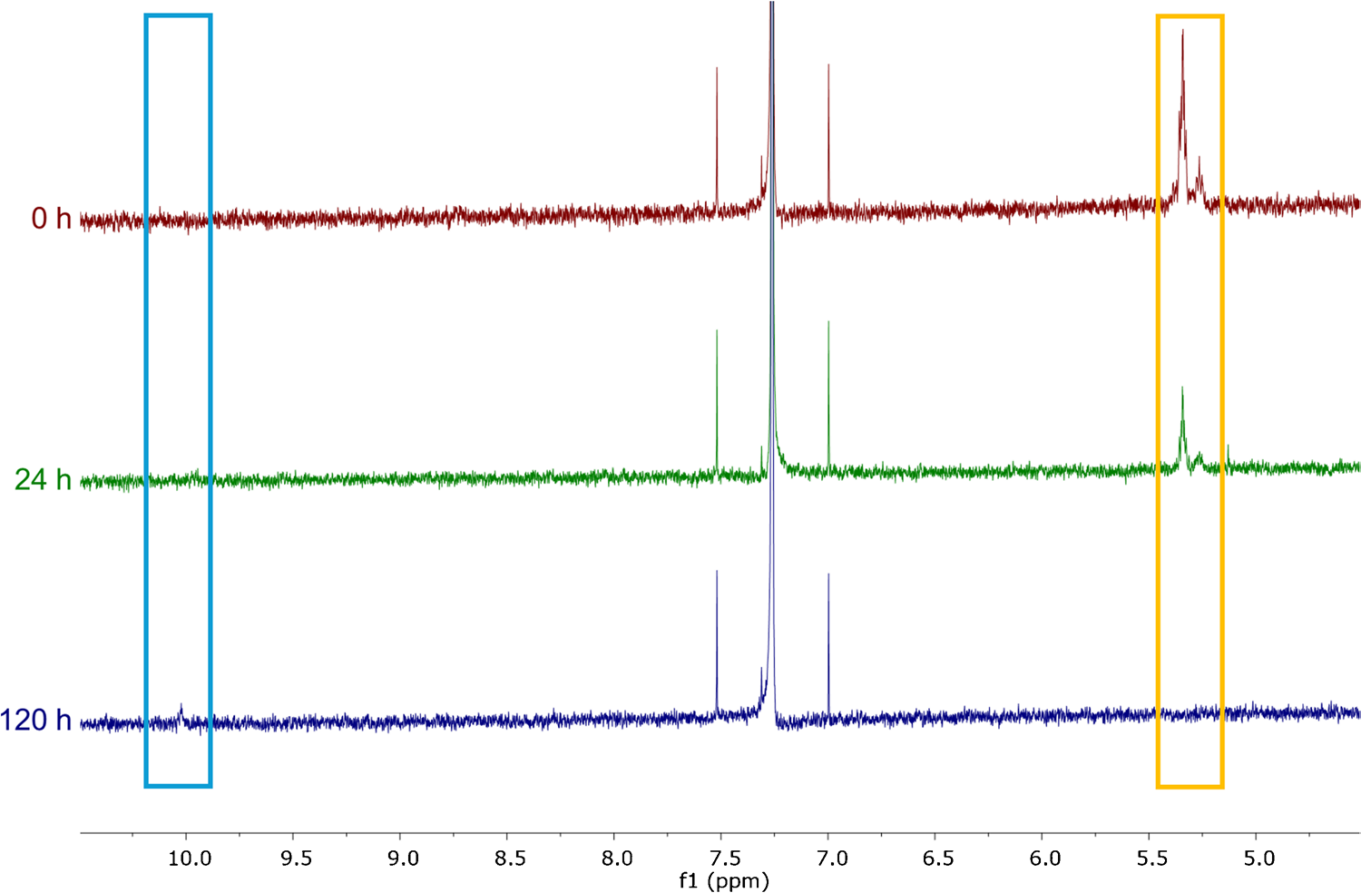
^1^H NMR spectra of glycerol trioleate after incubation for given times in the presence of ambient oxygen at a temperature of 40 °C. ^1^H NMR spectra are displayed in a range from 4.5 to 10.5 ppm. As a consequence of lipid oxidation, the signals for the olefinic protons (orange box, ~ 5.3 ppm) disappear over time, whereas aldehyde-based oxidation products are being formed (cyan box, ~ 10 ppm). Full spectra can be found in Supplementary Information

In order to demonstrate that the observed increase in fluorescence, as a consequence of a conversion of our artificial fluorogenic tracer (**1a**), is a valid surrogate for the lipid oxidation of the co-incubated native glycerol trioleate, we followed the oxidation of glycerol trioleate via ^1^H NMR spectroscopy. Consistent with the results from the fluorescence readout indicating a maximum oxidative conversion of our fluorogenic tracer (**1a**) after 120 h of incubation, we detected a nearly complete conversion of glycerol trioleate after 120 h of incubation via NMR (Fig. 6). This oxidative conversion of glycerol trioleate can nicely be followed in the ^1^H NMR spectra by the disappearing signals for the olefinic protons (~ 5.3 ppm) and the appearance of a new signal at ~ 10.0 ppm, which presumably shows the formation of aldehyde-based oxidation products since aldehyde protons are typically located in the area around 10 ppm. An intermediate oxidation of glycerol trioleate was detected after 24 h of incubation, which is also in line with the observations for the oxidative conversion of our fluorogenic tracer **1a** (Fig. 4B). Thus, the oxidative conversion of our fluorogenic tracer **1a** nicely reflects the lipid oxidation of the co-incubated glycerol trioleate and further demonstrates that our fluorogenic tracers are valuable tools for studying the dynamics of lipid oxidation.

## Conclusions

Herein, we report the design, synthesis and application of the first-in-class fluorogenic triacylglycerols that enable a dynamic monitoring of lipid oxidation via fluorescence readout. Our fluorogenic tracers were designed to resemble the structural and chemical properties of natural triacylglycerols as closely as possible and can be easily prepared from readily available starting materials, applying a straightforward synthesis procedure. A final-stage fluorescence labelling of azido-functionalized triacylglycerol precursors was achieved via Cu(I)-catalysed azide-alkyne cycloaddition. Due to the modularity of the established synthesis approach, the fatty acids or fluorophores can be easily exchanged. Our fluorogenic triacylglycerol **1a**, featuring two fluorescein-labelled mono-unsaturated fatty acid chains, enabled us to monitor lipid oxidation in an aqueous as well as lipid-based environment via fluorescent readout. Control experiments with the fluorogenic triacylglycerol **1b** with its fully saturated acyl chains show that **1b** and **1a** feature a highly similar hydrolytic stability, thus corroborating that a different behaviour of these tracers under oxidizing conditions can only be a consequence of lipid oxidation but not lipid hydrolysis. With this, we provided a first proof of principle for the application of fluorogenic lipids as molecular tools to monitor lipid oxidation, thereby highlighting the novelty of our approach as well as the difference to previously reported fluorogenic lipids that were only used as lipase substrates [8]. Moreover, a direct comparison of **1a** and **1b** under oxidizing conditions indicated that the sensitivity of our fluorescent tracers towards oxidation can be fine‐tuned by either increasing or decreasing the number of double bonds in the fluorescently labelled acyl chains. Since our experimental data for very long incubation times (≥ 5 days) indicated an oxidation of the fluorescein label, thus limiting the applicability of our tracers for long-term studies, our future investigations will be directed towards a replacement of the fluorescein label with alternative fluorophores featuring an enhanced stability towards oxidation [55, 56]. In summary, our fluorogenic substrates enabled a first important proof of principle demonstrating the applicability of fluorescently labelled triacylglycerols as tracers to monitor the dynamics of lipid oxidation, thus paving the way for novel discoveries in the field of lipid analytics.

## Supplementary Information

Below is the link to the electronic supplementary material.

Supplementary file1 (PDF 4571 KB)

## References

[1] Bordoni L, Petracci I, Zhao F, Min W, Pierella E, Assmann TS, Martinez JA, Gabbianelli R. Nutrigenomics of dietary lipids. Antioxidants. 2021;10:994. 10.3390/antiox10070994. 34206632 10.3390/antiox10070994 PMC8300813

[2] Savla R, Browne J, Plassat V, Wasan KM, Wasan EK. Review and analysis of FDA approved drugs using lipid-based formulations. Drug Dev Ind Pharm. 2017;43:1743–58. 10.1080/03639045.2017.1342654. 28673096 10.1080/03639045.2017.1342654

[3] Shahidi F, Zhong Y. Lipid oxidation and improving the oxidative stability. Chem Soc Rev. 2010;39:4067–79. 10.1039/b922183m. 20617249 10.1039/b922183m

[4] Vieira SA, Zhang G, Decker EA. Biological implications of lipid oxidation products. J Am Oil Chem Soc. 2017;94:339–51. 10.1007/s11746-017-2958-2.

[5] Schaich KM, Challenges in elucidating lipid oxidation mechanisms: when, where, and how do products arise? In: Logan A, Nienaber U, Pan X, editor. Lipid oxidation, Elsevier; 2013;pp. 1-52. 10.1016/B978-0-9830791-6-3.50004-7.

[6] Alireza SM, M. TS, Henriksson G, Johansson M. Effect of model lignin structures on the oxidation of unsaturated fatty acids. Polym Renewable Resour. 2010;1:69-90. 10.1177/204124791000100201.

[7] Dobarganes MC, Velasco J. Analysis of lipid hydroperoxides. Eur J Lipid Sci Technol. 2002;104:420–8. 10.1002/1438-9312(200207)104:7%3c420::AID-EJLT420%3e3.0.CO;2-N.

[8] Andersen RJ, Brask J. Synthesis and evaluation of fluorogenic triglycerides as lipase assay substrates. Chem Phys Lipids. 2016;198:72–9. 10.1016/j.chemphyslip.2016.05.007. 27241527 10.1016/j.chemphyslip.2016.05.007

[9] Cieplak P, Caldwell J, Kollman P. Molecular mechanical models for organic and biological systems going beyond the atom centered two body additive approximation: aqueous solution free energies of methanol and *N*-methyl acetamide, nucleic acid base, and amide hydrogen bonding and chloroform/water partition coefficients of the nucleic acid bases. J Comput Chem. 2001;22:1048–57. 10.1002/jcc.1065.

[10] Joung IS, Cheatham TE III. Determination of alkali and halide monovalent ion parameters for use in explicitly solvated biomolecular simulations. J Phys Chem B. 2008;112:9020–41. 10.1021/jp8001614. 18593145 10.1021/jp8001614 PMC2652252

[11] Sjöback R, Nygren J, Kubista M. Absorption and fluorescence properties of fluorescein. Spectrochim Acta, Part A. 1995;51:L7–21. 10.1016/0584-8539(95)01421-P.

[12] Le Guern F, Mussard V, Gaucher A, Rottman M, Prim D. Fluorescein derivatives as fluorescent probes for pH monitoring along recent biological applications. Int J Mol Sci. 2020;21:9217. 10.3390/ijms21239217. 33287208 10.3390/ijms21239217 PMC7729466

[13] Wang J, Wolf RM, Caldwell JW, Kollman PA, Case DA. Development and testing of a general amber force field. J Comput Chem. 2004;25:1157–74. 10.1002/jcc.20035. 15116359 10.1002/jcc.20035

[14] Wang J, Wang W, Kollman PA, Case DA. Automatic atom type and bond type perception in molecular mechanical calculations. J Mol Graph Model. 2006;25:247–60. 10.1016/j.jmgm.2005.12.005. 16458552 10.1016/j.jmgm.2005.12.005

[15] Cieplak P, Cornell WD, Bayly C, Kollman PA. Application of the multimolecule and multiconformational RESP methodology to biopolymers: charge derivation for DNA, RNA, and proteins. J Comput Chem. 1995;16:1357–77. 10.1002/jcc.540161106.

[16] Bayly CI, Cieplak P, Cornell W, Kollman PA. A well-behaved electrostatic potential based method using charge restraints for deriving atomic charges: the RESP model. J Phys Chem. 1993;97:10269–80. 10.1021/j100142a004.

[17] Frisch MJ, Trucks GW, Schlegel HB, Scuseria GE, Robb MA, Cheeseman JR, Scalmani G, Barone V, Petersson GA, Nakatsuji H, Li X, Caricato M, Marenich AV, Bloino J, Janesko BG, Gomperts R, Mennucci B, Hratchian HP, Ortiz JV, Izmaylov AF, Sonnenberg JL, Williams, Ding F, Lipparini F, Egidi F, Goings J, Peng B, Petrone A, Henderson T, Ranasinghe D, Zakrzewski VG, Gao J, Rega N, Zheng G, Liang W, Hada M, Ehara M, Toyota K, Fukuda R, Hasegawa J, Ishida M, Nakajima T, Honda Y, Kitao O, Nakai H, Vreven T, Throssell K, Montgomery Jr. JA, Peralta JE, Ogliaro F, Bearpark MJ, Heyd JJ, Brothers EN, Kudin KN, Staroverov VN, Keith TA, Kobayashi R, Normand J, Raghavachari K, Rendell AP, Burant JC, Iyengar SS, Tomasi J, Cossi M, Millam JM, Klene M, Adamo C, Cammi R, Ochterski JW, Martin RL, Morokuma K, Farkas O, Foresman JB, Fox DJ Gaussian 16 Rev. B.01, Wallingford, CT, 2016.

[18] Ditchfield R, Hehre WJ, Pople JA. Self-consistent molecular-orbital methods. IX. An extended Gaussian-type basis for molecular-orbital studies of organic molecules. J Chem Phys. 1971;54:724–8. 10.1063/1.1674902.

[19] Hehre WJ, Ditchfield R, Pople JA. Self-consistent molecular orbital methods. XII. Further extensions of Gaussian-type basis sets for use in molecular orbital studies of organic molecules. J Chem Phys. 1972;56:2257–61. 10.1063/1.1677527.

[20] Hariharan PC, Pople JA. Influence of polarization functions on molecular-orbital hydrogenation energies. Theor Chem Acc. 1973;28:213–22. 10.1007/BF00533485.

[21] Hariharan PC, Pople JA. Accuracy of AH equilibrium geometries by single determinant molecular-orbital theory. Mol Phys. 1974;27:209–14. 10.1080/00268977400100171.

[22] Gordon MS. The isomers of silacyclopropane. Chem Phys Lett. 1980;76:163–8. 10.1016/0009-2614(80)80628-2.

[23] Francl MM, Pietro WJ, Hehre WJ, Binkley JS, DeFrees DJ, Pople JA, Gordon MS. Self-consistent molecular orbital methods. 23. A polarization-type basis set for 2nd-row elements. J Chem Phys. 1982;77:3654–65. 10.1063/1.444267.

[24] Binning RC Jr, Curtiss LA. Compact contracted basis-sets for 3rd-row atoms - Ga-Kr. J Comput Chem. 1990;11:1206–16. 10.1002/jcc.540111013.

[25] Blaudeau J-P, McGrath MP, Curtiss LA, Radom L. Extension of Gaussian-2 (G2) theory to molecules containing third-row atoms K and Ca. J Chem Phys. 1997;107:5016–21. 10.1063/1.474865.

[26] Rassolov VA, Pople JA, Ratner MA, Windus TL. 6–31G* basis set for atoms K through Zn. J Chem Phys. 1998;109:1223–9. 10.1063/1.476673.

[27] Rassolov VA, Ratner MA, Pople JA, Redfern PC, Curtiss LA. 6–31G* basis set for third-row atoms. J Comput Chem. 2001;22:976–84. 10.1002/jcc.1058.

[28] Becke AD. Density-functional thermochemistry. III. The role of exact exchange. J Chem Phys. 1993;98:5648–52. 10.1063/1.464913.

[29] Mennucci B. Polarizable continuum model. Wiley Interdiscip Rev: Comput Mol Sci. 2012;2:386–404. 10.1002/wcms.1086.

[30] Tomasi J, Mennucci B, Cammi R. Quantum mechanical continuum solvation models. Chem Rev. 2005;105:2999–3094. 10.1021/cr9904009. 16092826 10.1021/cr9904009

[31] Cornell WD, Cieplak P, Bayly CI, Gould IR, Merz KM Jr, Ferguson DM, Spellmeyer DC, Fox T, Caldwell JW, Kollman PA. A second generation force field for the simulation of proteins and nucleic acids. J Am Chem Soc. 1995;117:5179–97. 10.1021/ja00124a002.

[32] Case DA, Aktulga HM, Belfon K, Ben-Shalom IY, Berryman JT, Brozell SR, Cerutti DS, Cheatham TE III, Cisneros GA, Cruzeiro VWD, Darden TA, Duke RE, Giambasu G, Gilson MK, Gohlke H, Goetz AW, Harris R, Izadi S, Izmailov SA, Kasavajhala K, Kaymak MC, King E, Kovalenko A, Kurtzman T, Lee TS, LeGrand S, Li P, Lin C, Liu J, Luchko T, Luo R, Machado M, Man V, Manathunga M, Merz KM, Miao Y, Mikhailovskii O, Monard G, Nguyen H, O’Hearn KA, Onufriev A, Pan F, Pantano S, Qi R, Rahnamoun A, Roe DR, Roitberg A, Sagui C, Schott-Verdugo S, Shajan A, Shen J, Simmerling CL, Skrynnikov NR, Smith J, Swails J, Walker RC, Wang J, Wang J, Wei H, Wolf RM, Wu X, Xiong Y, Xue Y, York DM, Zhao S. Kollman PA Amber 2022. San Francisco: University of California; 2022.

[33] Ryckaert J-P, Ciccotti G, Berendsen HJC. Numerical integration of the cartesian equations of motion of a system with constraints: molecular dynamics of *n*-alkanes. J Comput Phys. 1977;23:327–41. 10.1016/0021-9991(77)90098-5.

[34] Essmann U, Perera L, Berkowitz ML, Darden T, Lee H, Pedersen LG. A smooth particle mesh Ewald method. J Chem Phys. 1995;103:8577–93. 10.1063/1.470117.

[35] Darden T, York D, Pedersen L. Particle mesh Ewald: an N log(N) method for Ewald sums in large systems. J Chem Phys. 1993;98:10089–92. 10.1063/1.464397.

[36] Roe DR, Cheatham TE 3rd. PTRAJ and CPPTRAJ: software for processing and analysis of molecular dynamics trajectory data. J Chem Theory Comput. 2013;9:3084–95. 10.1021/ct400341p. 26583988 10.1021/ct400341p

[37] Van Rossum G, Drake FL, Python 3 Reference Manual. CreateSpace: Scotts Valley, CA, 2009.

[38] Hunter JD. Matplotlib: a 2D graphics environment. Comput Sci Eng. 2007;9:90–5. 10.1109/MCSE.2007.55.

[39] Kluyver T, Ragan-Kelley B, Pérez F, Granger B, Bussonnier M, Frederic J, Kelley K, Hamrick J, Grout J, Corlay S, Ivanov P, Avila D, Abdalla S, Willing C, Jupyter development team, Jupyter notebooks – a publishing format for reproducible computational workflows. In 20th International Conference on Electronic Publishing (01/01/16), Loizides, F.; Scmidt, B., Eds. IOS Press: 2016; pp 87-90.

[40] Humphrey W, Dalke A, Schulten K. VMD: visual molecular dynamics. J Mol Graph. 1996;14:33–8. 10.1016/0263-7855(96)00018-5. 8744570 10.1016/0263-7855(96)00018-5

[41] Beierlein FR, Paradas Palomo M, Sharapa DI, Zozulia O, Mokhir A, Clark T. DNA-dye-conjugates: conformations and spectra of fluorescence probes. PLoS ONE. 2016;11:e0160229. 10.1371/journal.pone.0160229. 27467071 10.1371/journal.pone.0160229 PMC4965132

[42] Ni G, Li Z, Liang K, Wu T, De Libero G, Xia C. Synthesis and evaluation of immunostimulant plasmalogen lysophosphatidylethanolamine and analogues for natural killer T cells. Bioorg Med Chem. 2014;22:2966–73. 10.1016/j.bmc.2014.04.012. 24767817 10.1016/j.bmc.2014.04.012

[43] Lisa M, Holcapek M. Characterization of triacylglycerol enantiomers using chiral HPLC/APCI-MS and synthesis of enantiomeric triacylglycerols. Anal Chem. 2013;85:1852–9. 10.1021/ac303237a. 23298510 10.1021/ac303237a

[44] Ragno D, Brandolese A, Urbani D, Di Carmine G, De Risi C, Bortolini O, Giovannini PP, Massi A. Esterification of glycerol and solketal by oxidative NHC-catalysis under heterogeneous batch and flow conditions. React Chem Eng. 2018;3:816–25. 10.1039/C8RE00143J.

[45] Kurzhals S, Zirbs R, Reimhult E. Synthesis and magneto-thermal actuation of iron oxide core-PNIPAM shell nanoparticles. ACS Appl Mater Interfaces. 2015;7:19342–52. 10.1021/acsami.5b05459. 26270412 10.1021/acsami.5b05459 PMC4559841

[46] Dougan H, Lyster DM, Vincent JS. Macrocyclic lactones as a source for radiohalogenated fatty acid analogs and their precursors. J Radioanal Nucl Chem. 1985;89:71–8. 10.1007/BF02070205.

[47] Pattipeiluhu R, Crielaard S, Klein-Schiphorst I, Florea BI, Kros A, Campbell F. Unbiased identification of the liposome protein corona using photoaffinity-based chemoproteomics. ACS Cent Sci. 2020;6:535–45. 10.1021/acscentsci.9b01222. 32342003 10.1021/acscentsci.9b01222 PMC7181318

[48] Brun MA, Tan KT, Griss R, Kielkowska A, Reymond L, Johnsson K. A semisynthetic fluorescent sensor protein for glutamate. J Am Chem Soc. 2012;134:7676–8. 10.1021/ja3002277. 22533301 10.1021/ja3002277

[49] Sjövali O, Kuksis A, Kallio H. Formation of triacylglycerol core aldehydes during rapid oxidation of corn and sunflower oils with tert-butyl hydroperoxide/Fe2+. Lipids. 2002;37:81–94. 10.1007/s11745-002-0867-5. 11876266 10.1007/s11745-002-0867-5

[50] Stryer L, Haugland RP. Energy transfer: a spectroscopic ruler. Proc Natl Acad Sci U S A. 1967;58:719–26. 10.1073/pnas.58.2.719. 5233469 10.1073/pnas.58.2.719 PMC335693

[51] Rostovtsev VV, Green LG, Fokin VV, Sharpless KB. A stepwise huisgen cycloaddition process: copper(I)-catalyzed regioselective “ligation” of azides and terminal alkynes. Angew Chem Int Ed Engl. 2002;41:2596–9. 10.1002/1521-3773(20020715)41:14%3c2596::AID-ANIE2596%3e3.0.CO;2-4. 12203546 10.1002/1521-3773(20020715)41:14<2596::AID-ANIE2596>3.0.CO;2-4

[52] Tornoe CW, Christensen C, Meldal M. Peptidotriazoles on solid phase: [1,2,3]-triazoles by regiospecific copper(I)-catalyzed 1,3-dipolar cycloadditions of terminal alkynes to azides. J Org Chem. 2002;67:3057–64. 10.1021/jo011148j. 11975567 10.1021/jo011148j

[53] Toy L, Huber ME, Schmidt MF, Weikert D, Schiedel M. Fluorescent ligands targeting the intracellular allosteric binding site of the chemokine receptor CCR2. ACS Chem Biol. 2022;17:2142–52. 10.1021/acschembio.2c00263. 35838163 10.1021/acschembio.2c00263

[54] Makrigiorgos GM, Kassis AI, Mahmood A, Bump EA, Savvides P. Novel fluorescein-based flow-cytometric method for detection of lipid peroxidation. Free Radic Biol Med. 1997;22:93–100. 10.1016/s0891-5849(96)00229-8. 8958133 10.1016/s0891-5849(96)00229-8

[55] Zheng Q, Juette MF, Jockusch S, Wasserman MR, Zhou Z, Altman RB, Blanchard SC. Ultra-stable organic fluorophores for single-molecule research. Chem Soc Rev. 2014;43:1044–56. 10.1039/c3cs60237k. 24177677 10.1039/c3cs60237k PMC3946787

[56] Zheng Q, Lavis LD. Development of photostable fluorophores for molecular imaging. Curr Opin Chem Biol. 2017;39:32–8. 10.1016/j.cbpa.2017.04.017. 28544971 10.1016/j.cbpa.2017.04.017

