# Supplementary material for: New fluorogenic triacylglycerols as sensors for dynamic measurement of lipid oxidation
Source: Anal Bioanal Chem. 2024 Nov 21;417(2):287–96. doi: 10.1007/s00216-024-05642-w (PMC11698881; doi:10.1007/s00216-024-05642-w)
Supplement: Supplementary file 1 — Supplementary file1 (PDF 4571 KB) [file 216_2024_5642_MOESM1_ESM.pdf]

Supporting Information

New fluorogenic triacylglycerols as sensors for dynamic measurement of lipid oxidation

Maria Handke,[1,2] Frank Beierlein,[3,4] Petra Imhof,[3,4] Matthias Schiedel[1,2,*] and Simon Hammann[1,5,6,*]

[1] Department of Chemistry and Pharmacy, Friedrich-Alexander-Universität Erlangen-Nürnberg, Nikolaus-Fiebiger-Straße 10, 91058 Erlangen, Germany

[2] Institute of Medicinal and Pharmaceutical Chemistry, Technische Universität Braunschweig, Beethovenstraße 55, 38106 Braunschweig, Germany

[3] Computer Chemistry Center, Friedrich-Alexander-Universität Erlangen-Nürnberg, Nägelsbachstraße 25, 91052 Erlangen, Germany

[4] Zentrum für Nationales Hochleistungsrechnen Erlangen (NHR@FAU), Martensstraße 1, 91058 Erlangen, Germany

[5] FAU NeW - Research Center for New Bioactive Compounds, Nikolaus-Fiebiger-Str. 10, 91058 Erlangen, Germany

[6] Department of Food Chemistry and Analytical Chemistry (170a), Institute of Food Chemistry, University of Hohenheim, Garbenstraße 28, 70599 Stuttgart, Germany

* Matthias Schiedel (matthias.schiedel@tu-braunschweig.de) and Simon Hammann (simon.hammann@uni-hohenheim.de) are co-corresponding authors of this manuscript.

Table of Contents

S3 Synthesis and Compound Characterization

S9 Supplementary Figures

S15 Supplementary Tables

S21 Supplementary NMR Spectra

S44 Supplementary HRMS Spectra

S46 Supplementary HPLC Chromatograms

S47 Supplementary References

Synthesis and Compound Characterization

General remarks regarding target compound synthesis.

The starting materials DL-1,2-isopropylideneglycerol (**2**), ω-6-hexadecenlactone (**5a**), hexadecanolactone (**5b**), and 5-carboxyfluorescein (**10**), were purchased from Sigma-Aldrich. Other chemicals (reagents, solvents) were purchased from different commercial suppliers (Abcr, Acros Organics, Alfa Aesar, BLDpharm, Sigma-Aldrich, TCI, Fisher chemical) and used without any further purification. Solvents were used in p.a. quality and dried according to common procedures, if necessary. Thin-layer chromatography (TLC) for reaction monitoring was performed with alumina plates coated with Merck silica gel 60 F254 (layer thickness: 0.2 mm) or Merck silica gel 60 RP-18 F254 (layer thickness: 0.2 mm) and analyzed under UV-light (254 nm and 366 nm). Compounds that could not be visualized with UV or fluorescence detection were derivatized with 0.05 % primuline and subsequently visualized at 366 nm. As an alternative method for reaction monitoring, we used high-performance liquid chromatography mass spectrometry (HPLC-MS). HPLC-MS analyses were performed using a Thermo Scientific Dionex UltiMate 3000 HPLC system in combination with a DAD detector (220/230/254 nm) and an Agilent ZORBAX ECLIPSE, XDB-C8 column (3.0 mm x 100 mm, 3.5 µm). Elution was performed at room temperature under gradient conditions. Eluent A was water containing 0.1% (*v*/*v*) formic acid; eluent B was methanol. Linear gradient conditions were as follows: 0-0.2 min: A=75%, B=25%; 0.2-6.0 min: linear increase to B=100%; 6.0-8.5 min: B=100%; 8.5-9.0 min: linear decrease to A=75%, B=25%; 9.0-12.0 min: A=75%, B=25%. A flow rate of 0.4 $mL\cdot min^{-1}$ was maintained during the entire elution. Mass detection was performed with a BRUKER amaZon SL mass spectrometer and using positive ESI. Flash column chromatography was performed with hand packed silica columns 60M (0.040-0.063 µm, 230-400 mesh) as a stationary phase on a Biotage Selekt automated flash purification system with UV-Vis detector. Yields were not optimized. NMR spectra were recorded using either a Bruker Avance 400 (^{1}H: 400 MHz, ^{13}C: 101 MHz) or Bruker Avance 600 (^{1}H: 600 MHz, ^{13}C: 151 MHz) instrument. The spectra are referenced against the residual NMR solvent signal and are reported as follows: ^{1}H: chemical shift δ (ppm), multiplicity (s = singlet, d = doublet, dd = doublet of doublets, t = triplet, m = multiplet, b = broad), coupling constant (*J* in Hz), integration. ^{13}C: chemical shift δ (ppm). Signals that are partially overlaid by a solvent signal are marked with an asterisk (*). High resolution mass spectra were measured with a timsTOF Pro Mass Spectrometer from Bruker Daltonics using positive ESI. HPLC analyses for compounds **1a** and **1b** were performed using an Agilent 1200 series HPLC system employing a diode array detector (DAD, detection at 210, 230, 250 or 254 nm). If not stated otherwise, the indicated purity was determined at a wavelength of 254 nm. We used a ZORBAX ECLIPSE, XDB-C8 column (4.6 mm x 150 mm, 5 µm) with a flow rate of 0.5 $mL\cdot min^{-1}$. Elution was performed at room temperature under gradient conditions. Eluent A was water containing 0.1% (*v*/*v*) TFA; eluent B was acetonitrile. Linear gradient conditions were as follows: 0-3.0 min: A=90%, B=10%; 3.0-18.0 min: linear increase to A=5%, B=95%; 18.0-24.0 min: A=5%, B=95%; 24.0-27.0 min: linear decrease to A=90%, B=10%; 27.0-30.0 min: A=90%, B=10%.

Individual synthesis procedures and compound characterization.

3-(Palmitoyloxy)propane-1,2-diyl (6E,6’E)bis(16-(4-((3‘,6‘-dihydroxy-3-oxo-3H-spiro[isobenzofuran-1,9‘-xanthene]-5-carboxamido)methyl)-1H-1,2,3-triazol-1-yl)hexadic-6-enoate) (**1a**, MAH16):

3-(Palmitoyloxy)propane-1,2-diyl (6*E*,6*E*’)-bis(16-azidohexadec-6-enoate) (**9a**; 11.0 mg, 12.4 µmol, 1.0 eq) and 3’,6’-dihydroxy-3-oxo-*N*-(prop-2-yn-1yl)-3*H*-spiro[isobenzofuran-1,9’-xanthene]-5-carboxamide (**11**; 11.3 mg, 27.3 µmol, 2.2 eq) were dissolved in 2 mL of EtOH. TBTA (1.32 mg, 2.49 µmol, 0.2 eq) was dissolved in 0.5 mL of DMF and added to the mixture. An aqueous $CuSO_4$ solution (0.1 M, 25 µL, 0.2 eq) and an aqueous solution of sodium ascorbate (0.1 M, 30 µL, 0.24 eq) were added in that order. The resulting reaction mixture was stirred for 3 h at 41 °C. The solvent was removed under reduced pressure. The obtained solid was purified by column chromatography (n-hexane/ethyl acetate (+ 1% HOAc), gradient: 35-100%). For the final purification pHPLC was used (acetonitrile/water (+ 0.1% TFA), gradient: 10-90%). The title compound was obtained as a yellow solid (11.2 mg, 53% yield). R_f = 0.30 (ethyl acetate (+ 1% HOAc)); ^{1}H NMR (400 MHz, DMSO-d_6, δ [ppm]): 10.15 (s, 4H), 9.23 (t, *J*=5.7 Hz, 2H), 8.18 (dd, *J*=8.0 Hz, 1.4 Hz, 2H), 8.06 (d, *J*=8.0 Hz,2H), 7.91 (s, 2H), 7.70 (t, *J*=1.0 Hz, 2H), 6.68 (d, *J*=2.2 Hz, 4H), 6.59-6.51 (m, 8H), 5.31 (t, *J*=3.8 Hz, 4H), 5.19-5.15 (m, 1H), 4.42 (d, *J*=5.5 Hz, 4H), 4.28-4.21 (m, 6H), 4.23 (d, *J*=5.5 Hz, 4H), 4.13-4.08 (m, 2H), 2.28-2.21 (m, 6H), 1.92-1.83 (m, 8H), 1.70 (p, *J*=7.1 Hz, 4H), 1.51-1.43 (s, 6H), 1.26-1.15 (m, 52H), 0.83-0.80 (m, 3H); ^{13}C NMR (DEPTQ, 151 MHz, DMSO-d_6, δ [ppm]): 172.44, 172.11, 167.97, 164.34, 159.58, 152.66, 151.80, 144.25, 140.29, 130.01, 129.97, 129.87, 129.42, 129.16, 128.25, 124.81, 122.84, 122.39, 112.68, 109.11, 102.21, 68.75, 61.75, 49.16, 34.93, 33.47, 33.34, 33.29, 31.92, 31.91, 31.78, 31.78, 31.26, 29.63, 29.01, 29.00, 28.97, 28.95, 28.93, 28.84, 28.78, 28.78, 28.67, 28.65, 28.53, 28.50, 28.38, 28.34, 28.33, 28.31, 27.83, 27.81, 25.63, 24.38, 24.35, 22.06, 13.90; HRMS m/z (ESI$^+$) [found: 856.4518; $C_{99}H_{124}N_8O_{18}^{2+}$ [M+2H]$^{2+}$ requires 858.4511]; HPLC retention time: 24.97 min (>99%).

3-(Palmitoyloxy)propane-1,2-diyl bis(16-(4-((3‘,6‘-dihydroxy-3-oxo-3H-spiro[isobenzofuran-1,9‘-xanthene]-5-carboxamido)methyl)-1H-1,2,3-triazol-1-yl)hexadecanoate) (**1b**, MAH12):

3-(Palmitoyloxy)propane-1,2-diyl bis(16-azidohexadecanoate) (**9b**; 20.0 mg, 22.5 µmol, 1.0 eq) and 3’,6’-dihydroxy-3-oxo-*N*-(prop-2-yn-1yl)-3*H*-spiro[isobenzofuran-1,9’-xanthene]-5-carboxamide (**11**; 18.6 mg, 45.0 µmol, 2.0 eq) were dissolved in 2 mL of EtOH. Tris[(1-benzyl-1*H*-1,2,3-triazol-4-yl)methyl]amin (TBTA, 2.39 mg, 4.51 µmol, 0.2 eq) was dissolved in 0.5 mL of DMF and added to the mixture. An aqueous $CuSO_4$ solution (0.1 M, 45 µL, 0.2 eq) and an aqueous solution of sodium ascorbate (0.1 M, 54 µL, 0.24 eq) were added in that order. The resulting reaction mixture was stirred for 3 h at 41 °C. The solvent was removed under reduced pressure. The obtained solid was purified by column chromatography (n-hexane/ethyl acetate (+ 1% HOAc), gradient: 35-100%). For the final purification pHPLC was used (acetonitrile/water (+ 0.1% TFA), gradient: 10-90%). The title compound was obtained as a yellow solid (22.6 mg, 58% yield). R_f = 0.28 (ethyl acetate (+ 1% HOAc)); ^{1}H NMR (400 MHz, DMSO-d_6, δ [ppm]): 10.16 (s, 4H), 9.24 (t, *J*=5.7 Hz, 2H), 8.19 (dd, *J*=8.0 Hz, 1.4 Hz, 2H), 8.07 (dd, *J*=8.0 Hz, 0.7 Hz, 2H), 7.91 (s, 2H), 7.71 (t, *J*=1.0 Hz, 2H), 6.68 (d, *J*=2.2 Hz, 4H), 6.60-6.52 (m, 8H), 5.21-5.15 (m, 1H), 4.42 (d, *J*=5.5 Hz, 4H), 4.30-4.21 (m, 6H), 4.15-4.08* (m, 2H), 2.29-2.22 (m, 6H), 1.76-1.67 (m, 4H), 1.53-1.44 (m, 6H), 1.24-1.14 (m, 66H), 0.85-0.76 (m, 3H); ^{13}C NMR (DEPTQ, 151 MHz, DMSO-d_6, δ [ppm]): 172.43, 172.10, 167.97, 164.34, 159.58, 152.66, 151.80, 144.24, 140.29, 129.41, 129.16, 128.25, 124.81, 122.84,

122.39, 112.68, 109.10, 102.21, 68.76, 61.75, 49.16, 34.93, 33.50, 33.35, 33.34, 31.26, 29.66, 29.02, 29.01, 28.98, 28.97, 28.92, 28.89, 28.86, 28.84, 28.73, 28.70, 28.68, 28.67, 28.37, 28.35, 28.34, 25.81, 24.41, 24.39, 22.06, 13.89; HRMS *m/z* (ESI^+) [found: 858.4671; $C_{99}H_{128}N_8O_{18}^{2+}$ $[M+2H]^{2+}$ requires 858.4668]; HPLC retention time: 26.60 min (> 99%).

2,3-Dihydroxypropyl palmitate (**4**):[1-3]

DL-1,2-Isopropylidenegylcerol (500 mg, 3.78 mmol, 1.00 eq), palmitic acid (999 mg, 3.90 mmol, 1.03 eq), DMAP (499 mg, 4.09 mmol, 1.08 eq), and DCC (749 mg, 3.63 mmol, 0.96 eq) were dissolved in 50 mL of CH_2Cl_2. The reaction mixture was incubated at ambient temperature for 1.5 h. Then, the formed solids were removed by filtration and the filter cake was washed with 30 mL of CH_2Cl_2. The filtrate was washed with 40 mL of 1 N HCl and afterwards with 40 mL saturated $NaHCO_3$ solution. The organic layer was dried with Na_2SO_4, filtered, and concentrated under reduced pressure to obtain (2,2-dimethyl-1,3-dioxolan-4-yl)methyl palmitate (**3**), which was used without any further purification for the subsequent reaction. **3** was dissolved in 250 mL of 80% acetic acid und stirred overnight at ambient temperature. Then, volatiles were removed under reduced pressure and the crude product (R_f= 0.30, acetone/isohexane: 1:3 (primulin staining)) was purified by flash column chromatography (acetone/isohexane, gradient: 10-40%). The title compound was obtained as a white solid (876 mg, 73% yield). ^1H NMR[a] (400 MHz, $CDCl_3$, δ [ppm]): 4.25-4.14 (m, 2H), 3.99-3.92 (m, 1H), 3.75-3.59 (m, 2H), 2.65 (bs, 1H), 2.37 (t, *J*=7.3 Hz, 2H), 2.23 (bs, 1H), 1.68-1.60 (m, 2H), 1.27 (m, 24H), 0.92-0.87 (m, 3H); LRMS: *m/z* (ESI^+) [found: 331; $C_{19}H_{39}O_4^+$ $[M+H]^+$ requires 331]. Obtained analytical data are in good agreement with literature values [1-3].

(E)-16-Hydroxyhexadec-6-enoic acid (**6a**):[4]

KOH (349 mg, 6.22 mmol, 1.57 eq) and ω-6-hexadecenlactone (**5a**; 1.00 g, 3.96 mmol, 1.0 eq) were dissolved in 12.5 mL of EtOH and stirred overnight at 80 °C. Then, water (30 mL) was added, and the solution was acidified (pH = 1-2) by the addition of 5 N H_2SO_4. The precipitate was separated by filtration, washed with water (60 mL), and dried under reduced pressure. The title compound was obtained as a white solid (1.01 g, 95% yield). ^1H NMR[a] (400 MHz, CD_3OD, δ [ppm]): 5.37-5.33 (m, 2H), 3.54 (t, *J*=6.7 Hz, 2H), 2.34-2.25 (m, 2H), 2.04-1.93 (m, 4H), 1.64-1.48 (m, 4H), 1.40-1.29 (m, 14H); ^{13}C NMR (DEPTQ, 101 MHz, CD_3OD, δ [ppm]): 176.02, 131.49, 62.98, 34.78, 33.65, 33.58, 30.76, 30.67, 30.21, 30.17, 30.05, 29.99, 26.80, 26.01; LRMS: *m/z* (ESI^+) [found: 271; $C_{16}H_{31}O_3^+$ $[M+H]^+$ requires 271].

[a] *Exchangeable protons of the hydroxyl group and the carboxylic acid could not be detected.*

16-Hydroxyhexadecanoic acid (**6b**):[4]

KOH (351 mg, 6.25 mmol, 1.59 eq) and hexadecanolactone (**5b**; 1.00 g, 3.93 mmol, 1.0 eq) were dissolved in 12.5 mL of EtOH and stirred overnight at 80 °C. Then, water (30 mL) was added, and the solution was acidified (pH = 1-2) by the addition of 5 N H_2SO_4. The precipitate was separated by filtration, washed with water (60 mL), and dried under reduced pressure. The title compound was obtained as a white solid (999.5 mg, 93% yield). ^1H NMR[a] (400 MHz, CD_3OD, δ [ppm]): 3.54 (t, *J*=6.6 Hz, 2H), 2.28 (t, *J*=7.4 Hz, 2H), 1.64-1.48 (m, 4H), 1.39-1.28 (m, 22H); ^{13}C NMR (DEPTQ, 101 MHz, CD_3OD, δ [ppm]): 177.70, 63.00, 34.95, 33.68, 30.79,

30.78, 30.76, 30.74, 30.63, 30.45, 30.26, 26.97, 26.11; LRMS: *m/z* (ESI^+) [found: 273; $C_{16}H_{33}O_3^+$ $[M+H]^+$ requires 272.4].

[a] *Exchangeable protons of the hydroxyl group and the carboxylic acid could not be detected.*

(E)-16-(Tosyloxy)hexadec-6-enoic acid (**7a**):[5]

4-Toluenesulfonyl chloride (1.21 g, 6.32 mmol, 4.5 eq) was added to a dried reaction flask filled with argon. Then, dry THF (2 mL) was added. (*E*)-16-Hydroxyhexadec-6-enoic acid (**6a**; 380 mg, 1.41 mmol, 1.0 eq) was dissolved in dry pyridine (2.83 mL) while cooling with an ice bath. Subsequently, the prepared solution of 4-toluenesulfonyl chloride was added in a dropwise manner to the solution of **6a** while cooling with an ice bath. Thereafter, the reaction mixture was stirred at ambient temperature for 3 h. After completion, the reaction was quenched with water (25 mL) and the aqueous layer was extracted with chloroform (3 x 30 mL). The combined organic layer was washed with 0.1 N H_2SO_4 (2 x 25 mL) and water (2 x 25 mL), dried over Na_2SO_4, filtered, and concentrated under reduced pressure. The crude product (R_f= 0.29, ethyl acetate/isohexane: 4:6 (primulin staining)) was purified by flash column chromatography (ethyl acetate/isohexane, gradient: 5-35%). The title compound was obtained as a white solid (213 mg, 35% yield). ^1H NMR (400 MHz, $CDCl_3$, δ [ppm]): 10.80 (bs, 1H), 7.82-7.76 (m, 2H), 7.37-7.32 (m, 2H), 5.38-5.32 (m, 2H), 4.01 (t, *J*=6.5 Hz, 2H), 2.45 (s, 3H), 2.34 (t, *J*=7.5 Hz, 2H), 1.98-1.89 (m, 4H), 1.67-1.57 (m, 4H), 1.35-1.24 (m, 14H); LRMS: *m/z* (ESI^+) [found: 447; $C_{23}H_{36}NaO_5S^+$ $[M+Na]^+$ requires 447].

16-(Tosyloxy)hexadecanoic acid (**7b**):[5]

4-Toluenesulfonyl chloride (1.42 g, 7.43 mmol, 4.5 eq) was added to a dried reaction flask filled with argon. Then, dry THF (2.5 mL) was added. 16-Hydroxyhexadecanoic acid (**6b**; 450 mg, 1.65 mmol, 1.0 eq) was dissolved in dry pyridine (3.33 mL) while cooling with an ice bath. Subsequently, the prepared solution of 4-toluenesulfonyl chloride was added, in a dropwise manner, to the solution of **6b** while cooling with an ice bath. Thereafter, the reaction mixture was stirred at ambient temperature for 3 h. After completion, the reaction was quenched with water (25 mL) and the aqueous layer was extracted with chloroform (3 x 30 mL). The combined organic layer was washed with 0.1 N H_2SO_4 (2 x 25 mL) and water (2 x 25 mL), dried over Na_2SO_4, filtered, and concentrated under reduced pressure. The crude product (R_f= 0.35, ethyl acetate/isohexane: 4:6 (primulin staining)) was purified by flash column chromatography (ethyl acetate/isohexane, gradient: 5-35%). The title compound was obtained as a white solid (311 mg, 44% yield). ^1H NMR (400 MHz, $CDCl_3$, δ [ppm]): 11.09 (bs, 1H), 7.82-7.76 (m, 2H), 7.38-7.31 (m, 2H), 4.01 (t, *J*=6.5 Hz, 2H), 2.45 (s, 3H), 2.35 (t, *J*=7.5 Hz, 2H), 1.66-1.58 (m, 4H), 1.29-1.21 (m, 22H); LRMS: *m/z* (ESI^+) [found: 449; $C_{23}H_{38}NaO_5S^+$ $[M+Na]^+$ requires 449].

(E)-16-Azidohexadec-6-enoic acid (**8a**):

Under an argon atmosphere *(E)*-16-(tosyloxy)hexadec-6-enoic acid (**7a**; 110 mg, 259 µmol, 1.0 eq) and sodium azide (50.5 mg, 777 µmol, 3.0 eq) were added to a dried microwave reaction tube. Then, extra dry DMF (3 mL) was added, and the reaction mixture was stirred overnight at 80 °C. Then, volatiles were removed under reduced pressure. The residue was dissolved in 0.5 M HCl (15 mL) and the aqueous layer was extracted with ethyl acetate (3 x 15

mL). The combined organic layer was concentrated under reduced pressure. The crude product (R_f= 0.10, ethyl acetate/cyclohexane: 1:4 (primulin staining)) was purified by flash column chromatography (ethyl acetate/cyclohexane: gradient: 5-25%). The title compound was obtained as a white solid (66.3 mg, 87% yield). ^{1}H NMR (400 MHz, $CDCl_3$, δ [ppm]): 10.84 (bs, 1H), 5.40-5.35 (m, 2H), 3.25 (t, *J*=7.0 Hz, 2H), 2.35 (t, *J*=7.5 Hz, 2H), 2.01-1.92 (m, 4H), 1.67-1.55 (m, 4H), 1.39-1.27 (m, 14H); LRMS: *m/z* (ESI^+) [found: 318; $C_{16}H_{29}N_3NaO_2^+$ $[M+Na]^+$requires 318].

16-Azidohexadecanoic acid (**8b**):[6]

Under an argon atmosphere 16-(tosyloxy)hexadecanoic acid (**7b**; 40.0 mg, 93.8 µmol, 1.0 eq) and sodium azide (18.3 mg, 281 µmol, 3.0 eq) were added to a dried microwave reaction tube. Then, extra dry DMF (1 mL) was added and the reaction mixture was stirred overnight at 80 °C. Then, volatiles were removed under reduced pressure. The residue was dissolved in 0.5 M HCl (15 mL) and the aqueous layer was extracted with ethyl acetate (3 x 15 mL). The combined organic layer was concentrated under reduced pressure. The crude product (R_f= 0.15, ethyl acetate/cyclohexane: 1:4 (primulin staining)) was purified by flash column chromatography (ethyl acetate/cyclohexane: gradient: 5-25%). The title compound was obtained as a white solid (12.3 mg, 44% yield). ^{1}H NMR (400 MHz, $CDCl_3$, δ [ppm]): 10.80 (bs, 1H), 3.25 (t, *J*=7.0 Hz, 2H), 2.35 (t, *J*=7.5 Hz, 2H), 1.70-1.54 (m, 4H), 1.45-1.16 (m, 22H); LRMS: *m/z* (ESI^+) [found: 320; $C_{16}H_{31}N_3NaO_2^+$ $[M+Na]^+$ requires 320].

3-(Palmitoyloxy)propane-1,2-diyl (6E,6'E)-bis(16-azidohexadec-6-enoate) (**9a**):

(E)-16-Azidohexadec-6-enoic acid (**8a**; 59.5 mg, 201 µmol, 2.2 eq) and 2,3-dihydroxypropyl palmitate (**4**; 30.0 mg, 90.8 µmol, 1.0 eq) were added to a dried microwave reaction tube. DCC (44.0 mg, 218 µmol, 2.4 eq) and DMAP (30.1 mg, 246 µmol, 2.7 eq) were added to a second microwave reaction tube and dissolved in 2 mL CH_2Cl_2. This solution was then added to the other microwave rection tube containing **4** and **8a**, and the reaction mixture was stirred overnight at ambient temperature. Subsequently, the reaction mixture was filtered, and the filter cake was washed with CH_2Cl_2 (30 mL). The filtrate was washed with 1 N HCl (25 mL) and saturated $NaHCO_3$ (25 mL). The organic layer was dried over Na_2SO_4, filtered, and concentrated under reduced pressure. The crude product was purified by flash column chromatography (ethyl acetate/isohexane, gradient: 0-50%). The title compound was obtained as a white solid (20.2 mg, 25% yield). R_f = 0.78 (ethyl acetate/isohexane: 1:9); ^{1}H NMR (400 MHz, $CDCl_3$, δ [ppm]): 5.44-5.32 (m, 4H), 5.30-5.22 (m, 1H), 4.29 (ddd, *J*=11.9 Hz, 4.3 Hz, 1.0 Hz, 2H), 4.14 (dd, *J*=11.9 Hz, 6.0 Hz, 2H), 3.25 (t, *J*=7.0 Hz, 4H), 2.35-2.27 (m, 6H), 2.03-1.88 (m, 8H), 1.70-1.51 (m, 12H), 1.42-1.16 (m, 50H), 0.91-0.85 (m, 3H); LRMS: *m/z* (ESI^+) [found: 886; $C_{51}H_{93}N_6O_6^+$ $[M+H]^+$ requires 886]

3-(Palmitoyloxy)propane-1,2-diyl bis(16-azidohexadecanoate) (**9b**):

16-Azidohexadecanoic acid (**8b**; 40.96 mg, 137.70 µmol, 2.22 eq) and 2,3-dihydroxypropylpalmitate (**4**; 20.50 mg, 62.03 µmol, 1.0 eq) were added to a dried microwave reaction tube. DCC (30.7 mg, 149 µmol, 2.4 eq) and DMAP (20.5 mg, 168 µmol, 2.7 eq) were added to a second microwave reaction tube and dissolved in 2 mL CH_2Cl_2. This

solution was then added to the other microwave rection tube containing **4** and **8b**, and the reaction mixture was stirred overnight at ambient temperature. Subsequently, the reaction mixture was filtered, and the filter cake was washed with CH_2Cl_2 (30 mL). The filtrate was washed with 1 N HCl (25 mL) and saturated $NaHCO_3$ (25 mL). The organic layer was dried over Na_2SO_4, filtered, and concentrated under reduced pressure. The crude product was purified by flash column chromatography (ethyl acetate/isohexane, gradient: 0-50%). The title compound was obtained as a white solid (25.5 mg, 46% yield). R_f = 0.67 (ethyl acetate/isohexane: 1:9); ^{1}H NMR (400 MHz, $CDCl_3$, δ [ppm]): 5.31-5.22 (m, 1H), 4.29 (dd, *J*=11.9 Hz, 4.3 Hz, 2H), 4.14 (dd, *J*=11.9 Hz, 6.0 Hz, 2H), 3.25 (t, *J*=7.0 Hz, 4H), 2.36-2.27 (m, 6H), 1.66-1.56 (m, 12H), 1.43-1.19 (m, 65H), 0.88 (t, *J*=6.6 Hz, 3H); LRMS: *m/z* (ESI^+) [found: 890.7; $C_{51}H_{97}N_6O_6^+$ $[M+H]^+$ requires 890]

3',6'-Dihydroxy-3-oxo-N-(prop-2-yn-1yl)-3H-spiro[isobenzofuran-1,9'-xanthene]-5-carboxamide (**11**):[7]

11, also referred to as 5-FAM-alkyne, was synthesized according to a previously reported procedure by Brun et al. [7]. In brief, fluorescein (**10**; 100 mg, 301 µmol, 1.0 eq), propargylamine (35.3 mg, 641 µmol, 2.1 eq) and HOBT (70.7 mg, 524 µmol, 1.7 eq) were dissolved in 3.5 mL of extra dry DMF. DIC (64.6 mg, 512 µmol, 1.7 eq) and TEA (52.4 mg, 518 µmol, 1.7 eq) were added and the mixture was stirred overnight at ambient temperature. Then, 1M HCl (10 mL) was added to the reaction mixture, followed by an extraction with DCM (3 x 15 mL). The combined organic layer was washed with brine (2 x 25 mL), dried over Na_2SO_4, filtered, and concentrated under reduced pressure. The crude product was purified by column chromatography (DCM/methanol; gradient: 2-10%). The title compound was obtained as a yellow solid (86 mg, 41% yield). R_f = 0.36 (DCM/methanol: 9:1); LRMS: *m/z* (ESI^+) [found: 414; $C_{24}H_{16}NO_6^+$ $[M+H]^+$ requires 414]. Obtained analytical data are in good agreement with literature values [7].

Supplementary Figures

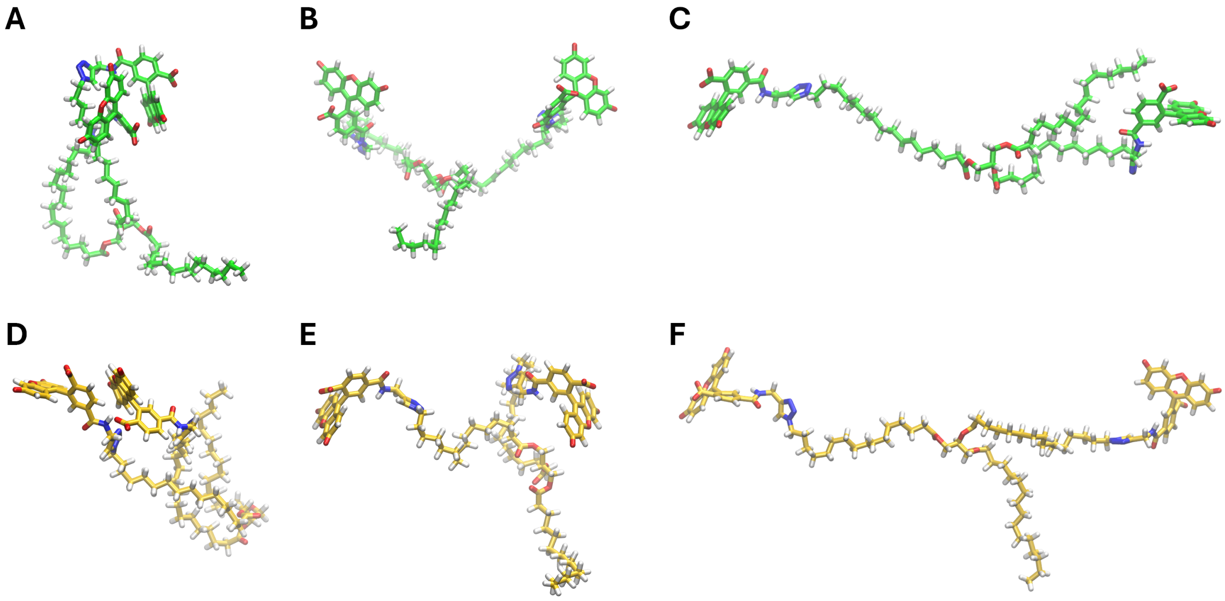

Figure S1. Snapshots of predicted conformations of **1a** (green) and **1b** (yellow) with small (A, D), medium (B, E), and large (C, F) distances between their fluorescein tags.

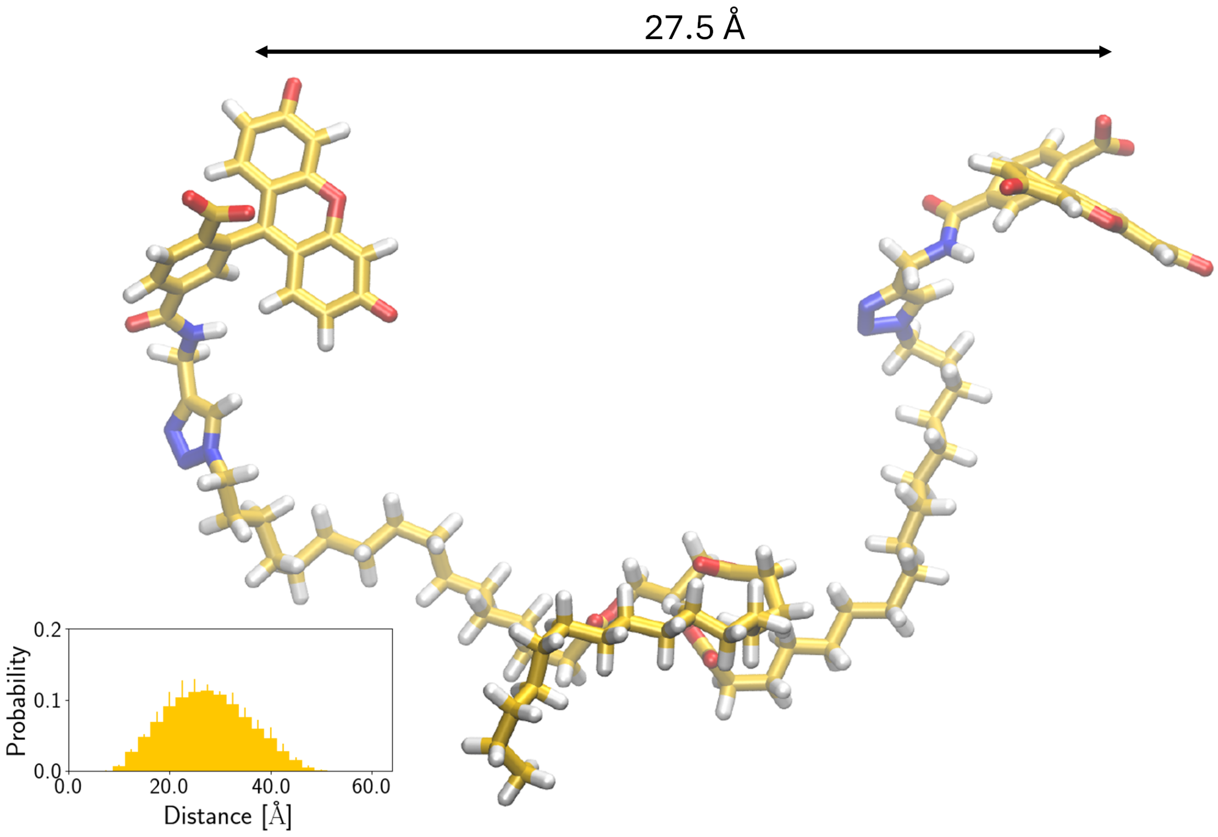

Figure S2. Medoid conformation of **1b** as predicted by MD simulations. Inset shows the predicted distribution of distances between the two fluorophore labels of **1b**.

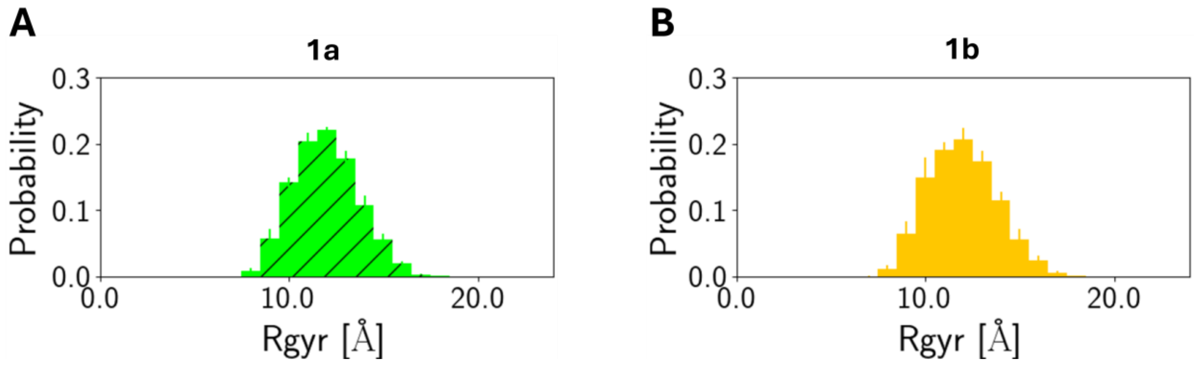

Figure S3. Fluorogenic triacylglycerols **1a** (A) and **1b** (B) are highly similar regarding their predicted distributions of radii of gyration. The radius of gyration is a measure for the compactness of a molecule.

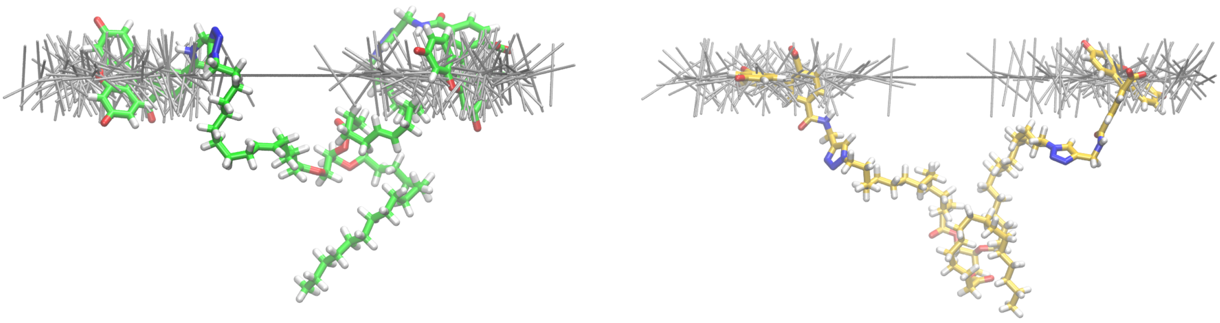

Figure S4. Predicted medoid structures and transition dipole vectors (silver "needles") of the fluorescent labels of **1a** (geen) or **1b** (yellow), respectively. The darker line indicates the distance between the xanthene rings in the medoid structures. For clarity only every 100th frame is shown.

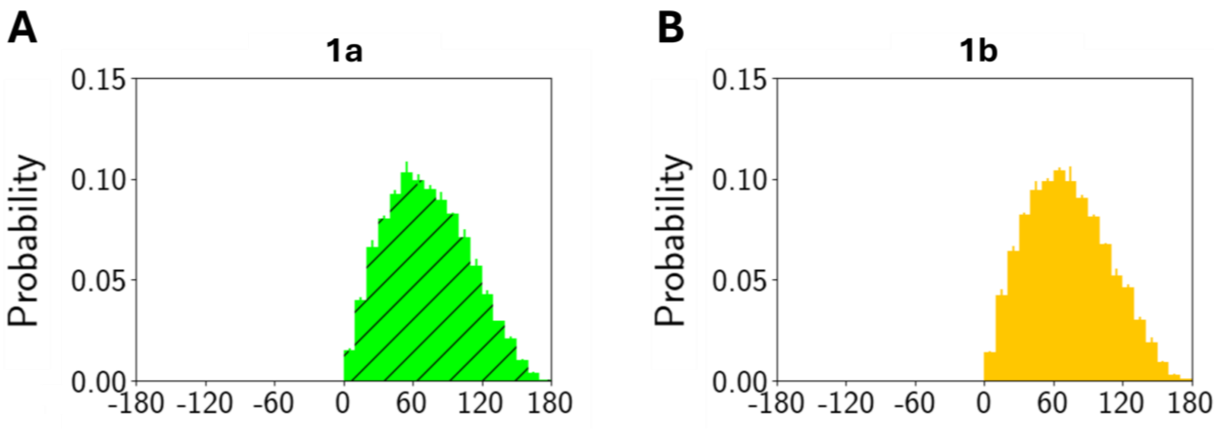

Figure S5. Fluorogenic triacylglycerols **1a** (A) and **1b** (B) are highly similar regarding the predicted distributions of the angle between the normal vectors of the xanthene ring planes of their two fluorophore labels.

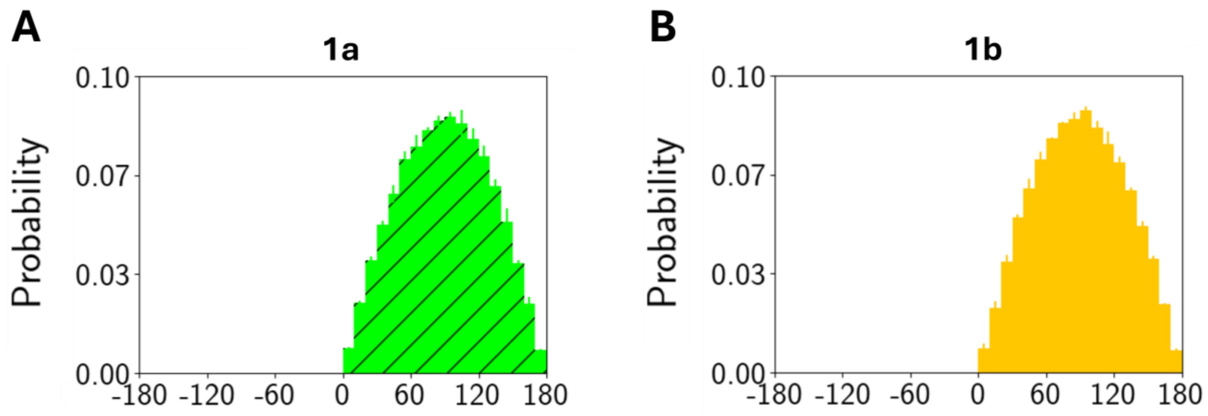

Figure S6. Fluorogenic triacylglycerols **1a** (A) and **1b** (B) are highly similar regarding the predicted distributions of the angle between the transition dipole vectors of their two fluorophore labels.

Supplementary Tables

Table S1A. Fluorescence signals over time for compound **1a** in H_2O/EtOH (1:9 (V/V)) at 80 °C (first triplicate measurement).

Time [min]	Fluorescence [RFU]			Fluorescence mean [RFU]
0.00	16.12	14.86	13.33	14.77
2.00	13.05	12.66	12.93	12.88
5.00	14.05	13.61	13.04	13.57
10.0	14.26	13.79	12.46	13.50
20.0	16.35	17.31	16.93	16.86
40.0	17.54	17.71	18.12	17.79
60.0	20.98	21.00	20.97	20.98
90.0	22.60	23.31	24.41	23.44

Table S1B. Fluorescence signals over time for compound **1a** in H_2O/EtOH (1:9 (V/V)) at 80 °C (second triplicate measurement).

Time [min]	Fluorescence [RFU]			Fluorescence mean [RFU]
0.00	30.94	29.43	28.75	29.71
2.00	27.45	28.12	28.75	28.11
5.00	31.67	30.53	29.64	30.61
10.0	30.96	31.84	32.89	31.90
20.0	34.61	32.84	32.89	33.45
40.0	37.83	36.25	36.96	37.01
60.0	40.61	37.42	38.98	39.00
90.0	41.86	40.71	39.54	40.70

Table S1C. Fluorescence signals over time for compound **1a** in H_2O/EtOH (1:9 (V/V)) at 80 °C (third triplicate measurement).

Time [min]	Fluorescence [RFU]			Fluorescence mean [RFU]
0.00	22.38	22.70	21.00	22.03
2.00	21.53	20.41	19.92	20.62
5.00	22.87	21.44	20.19	21.50
10.0	23.11	22.39	22.75	22.75
20.0	27.77	26.69	25.21	26.56
40.0	30.81	30.06	29.42	30.10
60.0	32.08	31.21	30.85	31.38
90.0	33.80	33.59	34.11	33.83

Table S2A. Fluorescence signals over time for compound **1a** in KOH (0.89 M), H_2O/EtOH (1:9 (V/V)) at 80 °C (first triplicate measurement).

Time [min]	Fluorescence [RFU]			Fluorescence mean [RFU]
0.00	307.5	306.8	307.0	307.1
2.00	318.4	317.5	316.2	317.4
5.00	326.3	325.6	325.0	325.7
10.0	349.3	348.7	349.5	349.2
20.0	353.8	354.9	353.9	354.2
40.0	356.7	356.3	356.2	356.4
60.0	362.6	361.6	361.9	362.0
90.0	363.8	364.1	364.0	364.0

Table S2B. Fluorescence signals over time for compound **1a** in KOH (0.89 M), H_2O/EtOH (1:9 (V/V)) at 80 °C (second triplicate measurement).

Time [min]	Fluorescence [RFU]			Fluorescence mean [RFU]
0.00	222.8	223.4	223.3	223.2
2.00	231.8	231.6	230.2	231.2
5.00	246.2	246.5	245.8	246.2
10.0	261.2	260.5	259.5	260.4
20.0	280.1	276.7	288.5	281.8
40.0	306.1	306.0	305.7	305.9
60.0	310.6	310.6	309.6	310.3
90.0	311.1	311.3	311.0	311.1

Table S2C. Fluorescence signals over time for compound **1a** in KOH (0.89 M), H_2O/EtOH (1:9 (V/V)) at 80 °C (third triplicate measurement).

Time [min]	Fluorescence [RFU]			Fluorescence mean [RFU]
0.00	255.4	256.5	256.9	256.2
2.00	262.1	261.4	261.0	261.5
5.00	277.7	275.6	277.0	276.8
10.0	290.1	289.1	289.4	289.6
20.0	307.2	308.4	309.0	308.2
40.0	315.8	315.3	316.1	315.7
60.0	330.9	331.2	331.2	331.1
90.0	332.9	332.5	331.9	332.4

Table S3A. Fluorescence signals over time for compound **1b** in H_2O/EtOH (1:9 (V/V)) at 80 °C (first triplicate measurement).

Time [min]	Fluorescence [RFU]			Fluorescence mean [RFU]
0.00	10.66	10.40	10.33	10.46
2.00	10.32	9.94	9.67	9.98
5.00	10.82	10.26	10.21	10.43
10.0	9.59	9.94	9.67	9.73
20.0	10.12	11.57	9.83	10.51
40.0	14.55	14.34	15.10	14.66
60.0	16.95	16.00	15.95	16.30
90.0	18.29	18.67	19.46	18.81

Table S3B. Fluorescence signals over time for compound **1b** in H_2O/EtOH (1:9 (V/V)) at 80 °C (second triplicate measurement).

Time [min]	Fluorescence [RFU]			Fluorescence mean [RFU]
0.00	14.52	13.84	12.21	13.52
2.00	11.21	12.79	11.10	11.70
5.00	13.73	12.94	11.80	12.82
10.0	15.56	14.83	14.33	14.91
20.0	16.99	17.66	17.20	17.28
40.0	18.74	17.46	17.03	17.74
60.0	24.26	23.71	23.53	23.83
90.0	28.37	30.55	29.28	29.40

Table S3C. Fluorescence signals over time for compound **1b** in H_2O/EtOH (1:9 (V/V)) at 80 °C (third triplicate measurement).

Time [min]	Fluorescence [RFU]			Fluorescence mean [RFU]
0.00	15.43	16.99	17.42	16.61
2.00	14.41	13.73	14.12	14.09
5.00	15.38	14.54	13.88	14.60
10.0	16.78	15.49	15.09	15.79
20.0	17.55	18.06	16.24	17.28
40.0	20.75	19.41	18.88	19.68
60.0	21.64	22.16	23.90	22.57
90.0	23.44	24.89	25.37	24.57

Table S4A. Fluorescence signals over time for compound **1b** in KOH (0.89 M), H_2O/EtOH (1:9 (V/V)) at 80 °C (first triplicate measurement).

Time [min]	Fluorescence [RFU]			Fluorescence mean [RFU]
0.00	193.2	192.3	190.8	192.1
2.00	197.8	197.5	197.4	197.5
5.00	203.2	202.6	203.0	202.9
10.0	207.7	207.9	207.8	207.8
20.0	230.2	230.4	230.2	230.3
40.0	293.4	292.6	293.4	293.2
60.0	292.6	293.4	292.7	292.9
90.0	295.5	295.8	295.9	295.7

Table S4B. Fluorescence signals over time for compound **1b** in KOH (0.89 M), H_2O/EtOH (1:9 (V/V)) at 80 °C (second triplicate measurement).

Time [min]	Fluorescence [RFU]			Fluorescence mean [RFU]
0.00	221.8	220.8	220.6	221.0
2.00	226.8	226.0	225.4	226.1
5.00	233.4	232.5	231.3	232.4
10.0	256.4	255.5	255.2	255.7
20.0	290.0	289.3	288.5	289.3
40.0	300.5	302.5	301.8	301.6
60.0	299.9	300.6	300.7	300.4
90.0	299.7	298.7	299.3	299.2

Table S4C. Fluorescence signals over time for compound **1b** in KOH (0.89 M), H_2O/EtOH (1:9 (V/V)) at 80 °C (third triplicate measurement).

Time [min]	Fluorescence [RFU]			Fluorescence mean [RFU]
0.00	205.4	204.2	203.1	204.3
2.00	217.6	216.4	215.7	216.6
5.00	227.3	225.7	224.1	225.7
10.0	246.3	245.1	244.2	245.2
20.0	260.4	259.0	258.2	259.2
40.0	280.9	281.8	280.1	280.9
60.0	283.6	284.8	283.4	283.9
90.0	283.9	283.3	281.6	282.9

Table S5. Fluorescence signals over time for compound **1a** during oxidation in an aqueous environment.

Time [h]	Fluorescence [RFU]			Fluorescence mean [RFU]
0.00	3.51	3.86	3.68	3.68
1.00	5.33	5.06	4.90	5.10
18.0	11.93	11.12	11.07	11.37
24.0	20.06	19.90	19.92	19.96
42.0	24.31	23.97	24.03	24.10
66.0	26.45	26.37	26.21	26.34
168	27.30	27.23	27.19	27.24
336	23.18	23.11	23.05	23.11

Table S6. Fluorescence signals over time for compound **1b** during oxidation in an aqueous environment.

Time [h]	Fluorescence [RFU]			Fluorescence mean [RFU]
0.00	0.33	0.39	0.42	0.38
1.00	0.94	0.89	0.78	0.87
18.0	1.61	1.75	1.67	1.68
24.0	1.88	1.87	1.77	1.84
42.0	1.99	2.07	2.05	2.04
66.0	2.24	2.11	1.98	2.11
168	2.87	2.75	2.81	2.81
336	0.92	0.90	0.87	0.90

Table S7A. Fluorescence signals over time for compound **1a** during oxidation in a lipid-based environment (first triplicate measurement).

Time [h]	Fluorescence [RFU]			Fluorescence mean [RFU]
0.00	29.27	29.41	29.16	29.28
24.0	251.2	250.5	250.1	250.6
120	450.2	450.5	448.9	449.9
264	282.7	281.7	281.2	281.9

Table S7B. Fluorescence signals over time for compound **1a** during oxidation in a lipid-based environment (second triplicate measurement).

Time [h]	Fluorescence [RFU]			Fluorescence mean [RFU]
0.00	27.07	26.93	26.72	26.91
24.0	207.5	209.8	209.6	209.0
120	380.1	380.6	380.0	380.2
264	229.2	230.7	228.4	229.4

Table S7C. Fluorescence signals over time for compound **1a** during oxidation in a lipid-based environment (third triplicate measurement).

Time [h]	Fluorescence [RFU]			Fluorescence mean [RFU]
0.00	32.54	32.39	32.20	32.38
24.0	319.2	318.7	318.3	318.7
120	534.1	533.1	531.2	532.8
264	312.3	310.7	309.5	310.8

Table S8A. Fluorescence signals over time for compound **1b** during oxidation in a lipid-based environment (first triplicate measurement).

Time [h]	Fluorescence [RFU]			Fluorescence mean [RFU]
0.00	5.24	5.37	5.18	5.26
24.0	57.67	57.04	56.51	57.07
120	115.9	114.9	113.8	114.89
264	66.60	64.98	64.92	65.50

Table S8B. Fluorescence signals over time for compound **1b** during oxidation in a lipid-based environment (second triplicate measurement).

Time [h]	Fluorescence [RFU]			Fluorescence mean [RFU]
0.00	4.98	4.81	4.76	4.85
24.0	47.83	47.57	47.52	47.64
120	93.60	93.07	93.09	93.25
264	51.62	50.92	50.05	50.86

Table S8C. Fluorescence signals over time for compound **1b** during oxidation in a lipid-based environment (third triplicate measurement).

Time [h]	Fluorescence [RFU]			Fluorescence mean [RFU]
0.00	2.65	2.53	2.60	2.59
24.0	35.59	35.36	35.31	35.42
120	82.84	83.49	83.33	83.22
264	49.10	48.93	48.80	48.94

Supplementary NMR Spectra

NMR spectra of the synthesized compounds **1a**-**b**, **4**, **6a**-**b**, **7a**-**b**, **8a**-**b**, **9a**-**b** and glycerol trioleate after 0 h, 24 h, and 120 h of incubation in the presence of ambient oxygen at 40 °C can be found on the following pages.

^{1}H NMR (400 MHz, DMSO-d_6) for compound **1a** (MAH16)

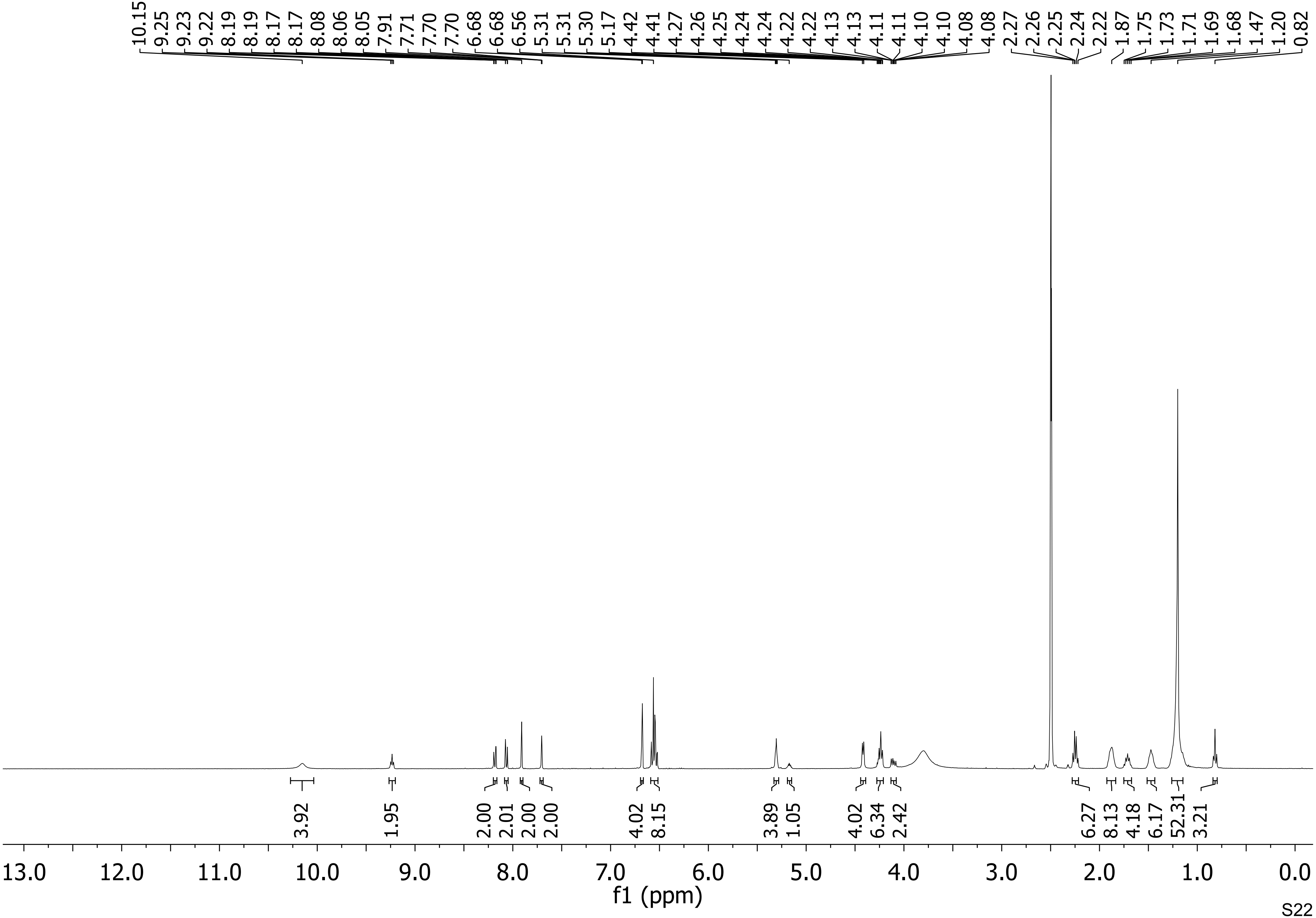

^{13}C NMR (DEPTQ, 151 MHz, DMSO-d_6) for compound **1a** (MAH16) full view

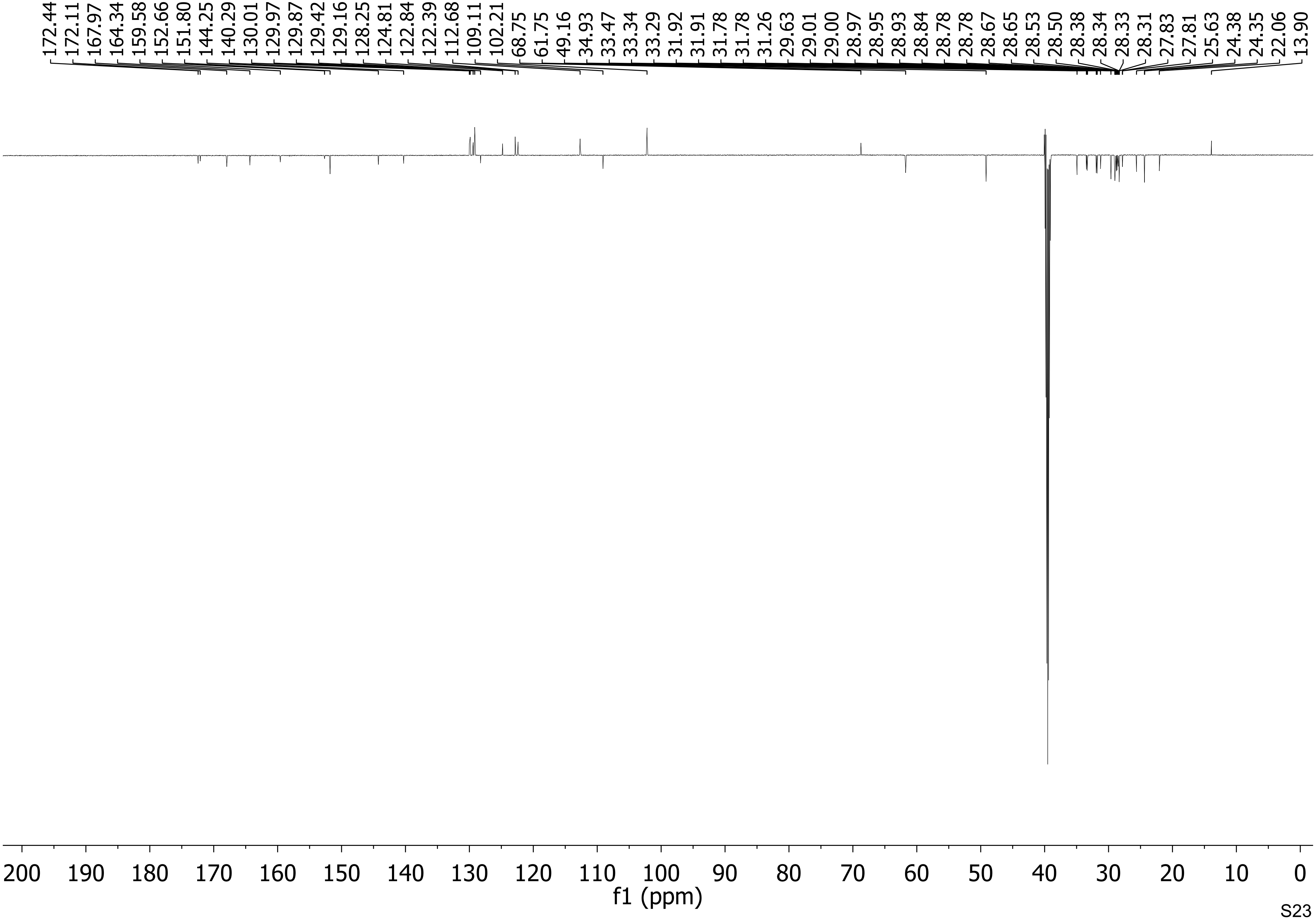

^{13}C NMR (DEPTQ, 151 MHz, DMSO-d_6) for compound **1a** (MAH16) zoomed-in view 1

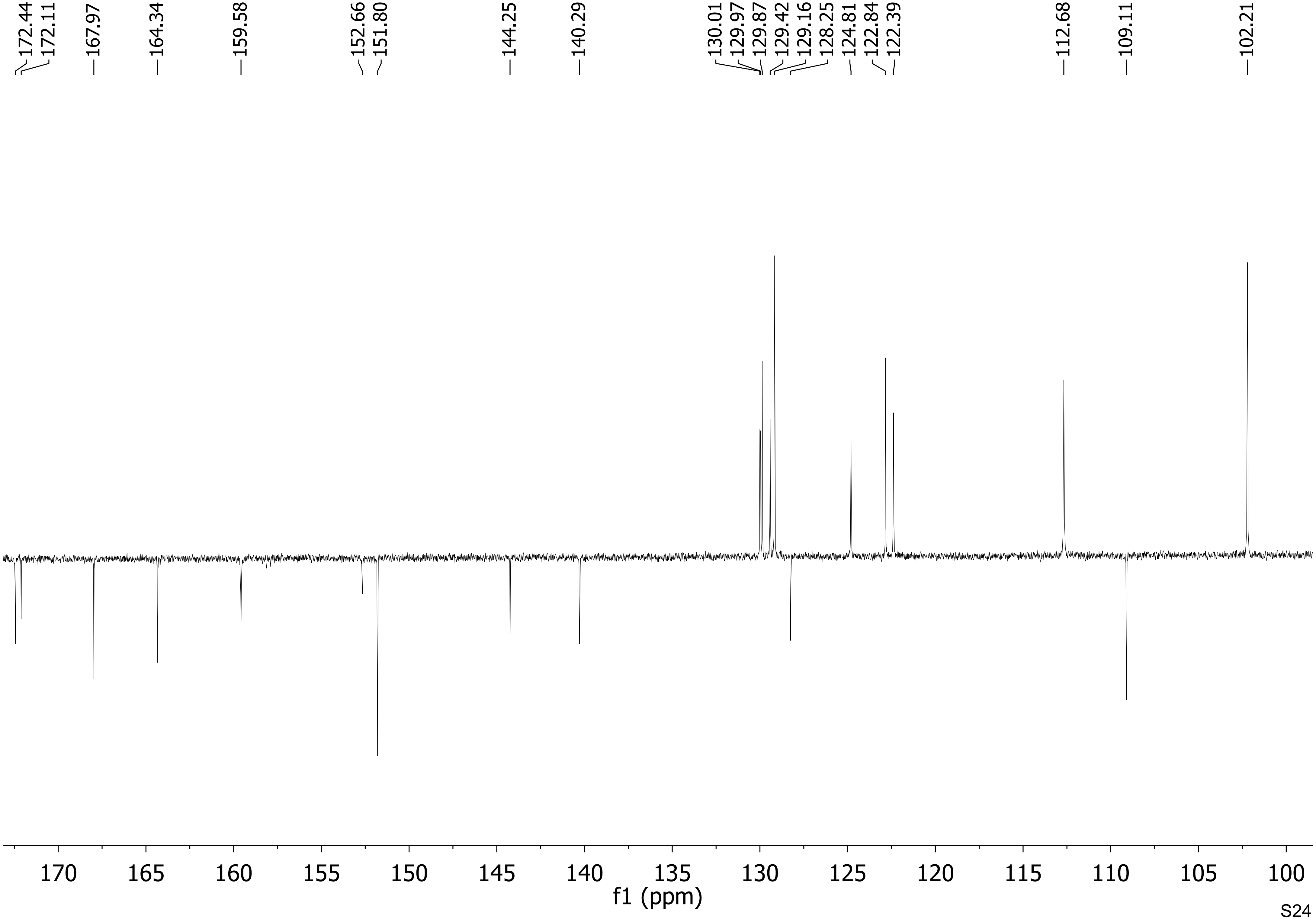

^{13}C NMR (DEPTQ, 151 MHz, DMSO-d_6) for compound **1a** (MAH16) zoomed-in view 2

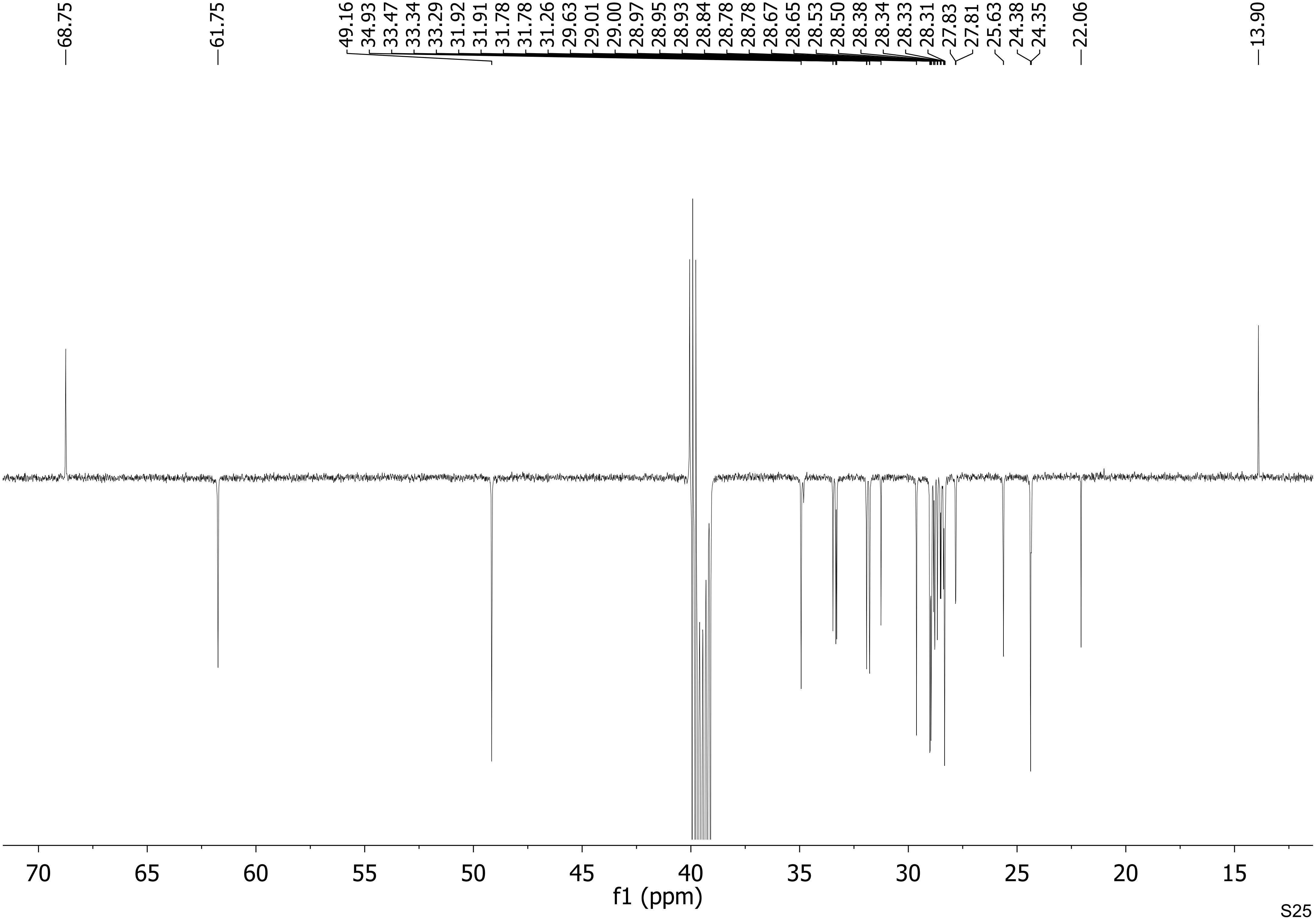

^{1}H NMR (400 MHz, DMSO-d_6) for compound **1b** (MAH12)

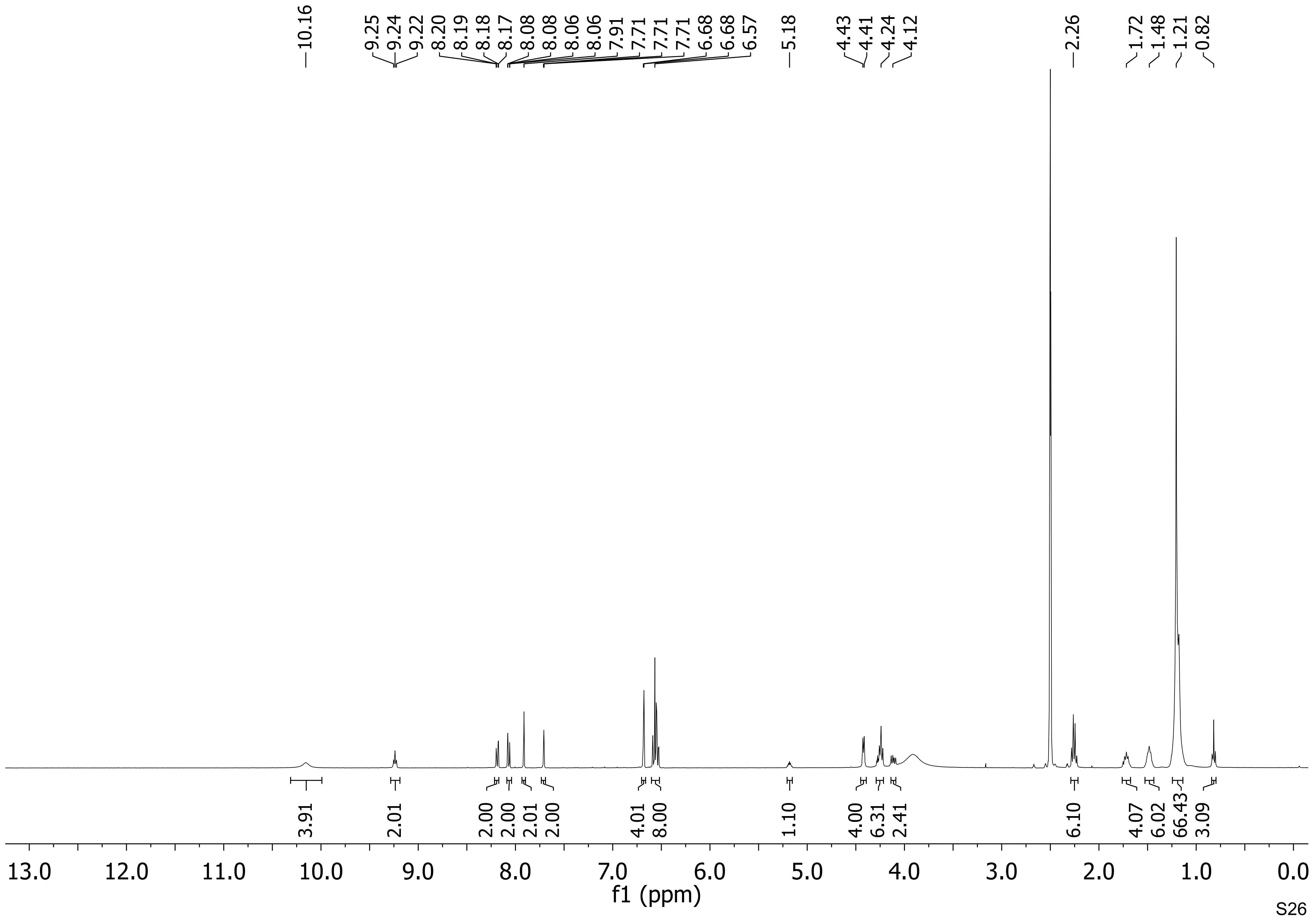

^{13}C NMR (DEPTQ, 151 MHz, DMSO-d_6) for compound **1b** (MAH12) full view

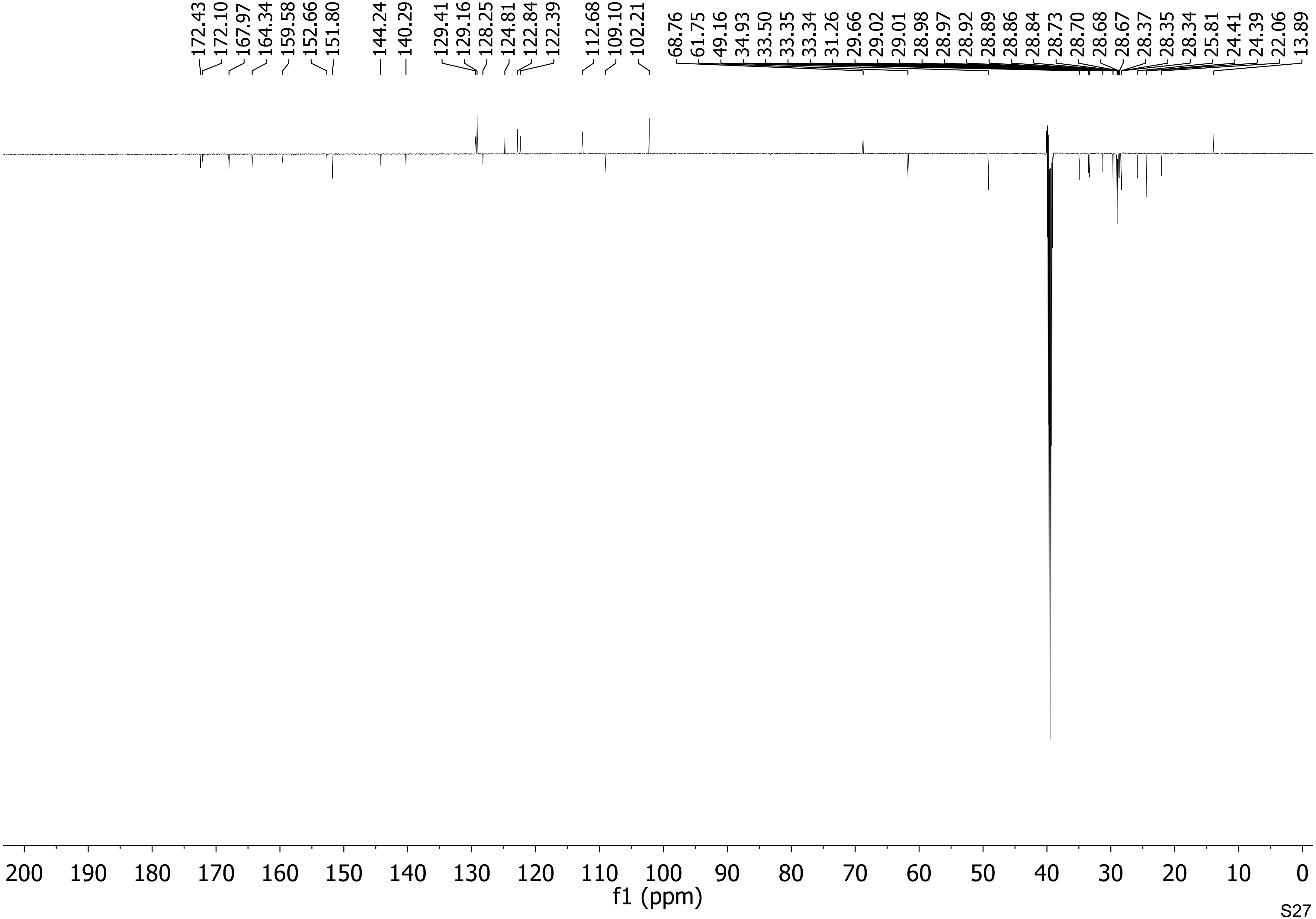

^{13}C NMR (DEPTQ, 151 MHz, DMSO-d_6) for compound **1b** (MAH12) zoomed-in view 1

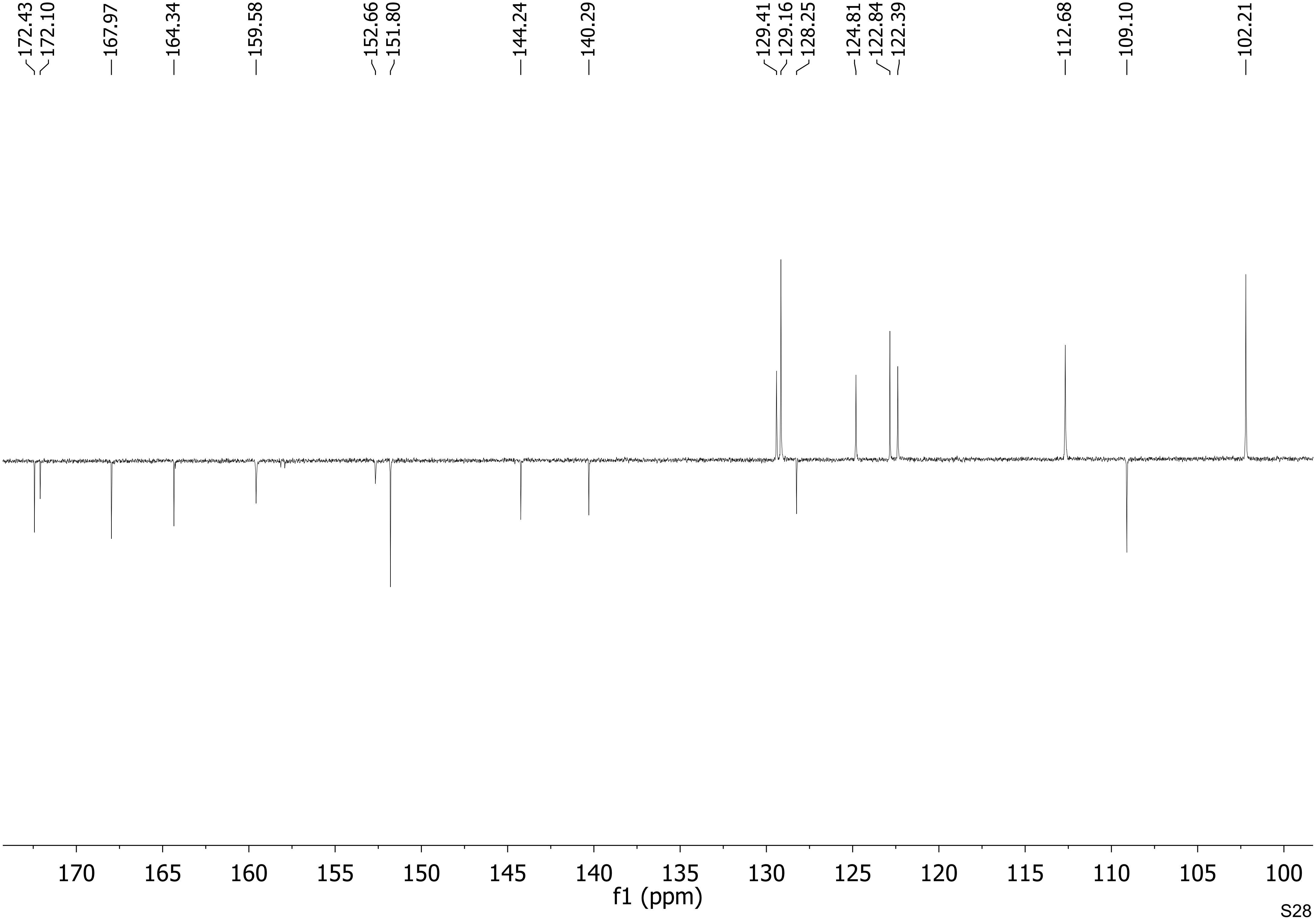

^{13}C NMR (DEPTQ, 151 MHz, DMSO-d_6) for compound **1b** (MAH12) zoomed-in view 2

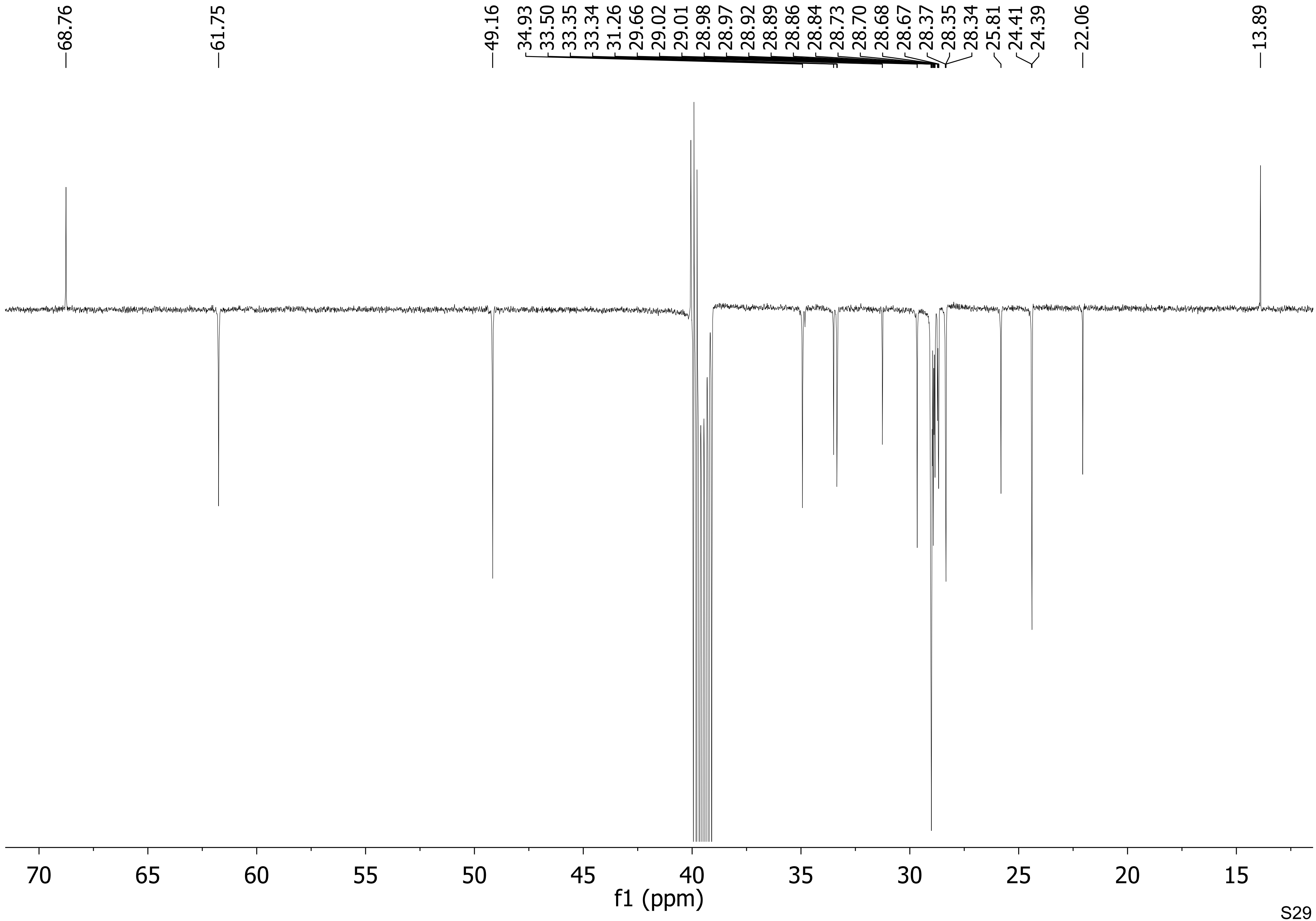

^1H NMR (400 MHz, $CDCl_3$) for compound **4**

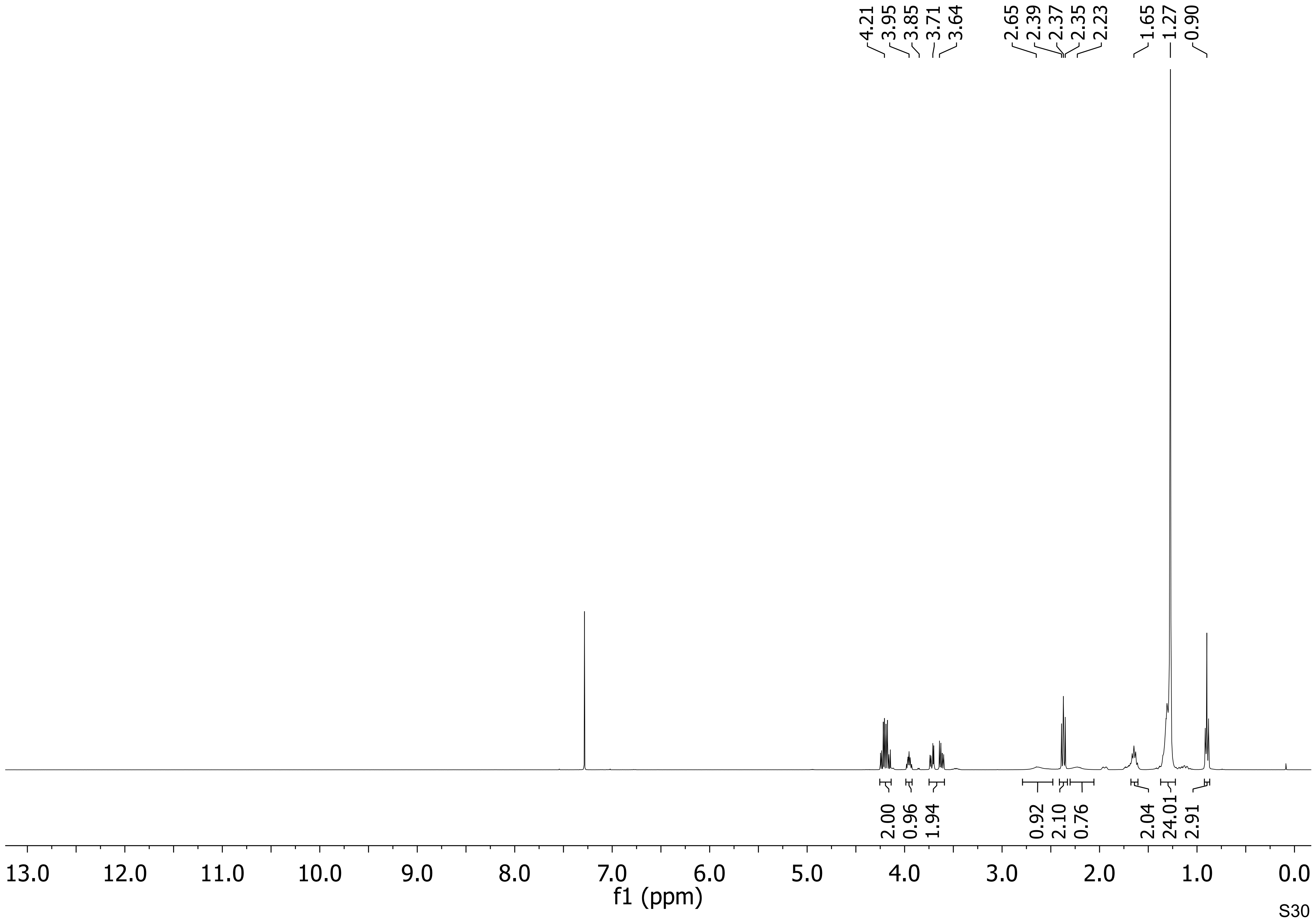

^{1}H NMR (400 MHz, CD_3OD) for compound **6a**

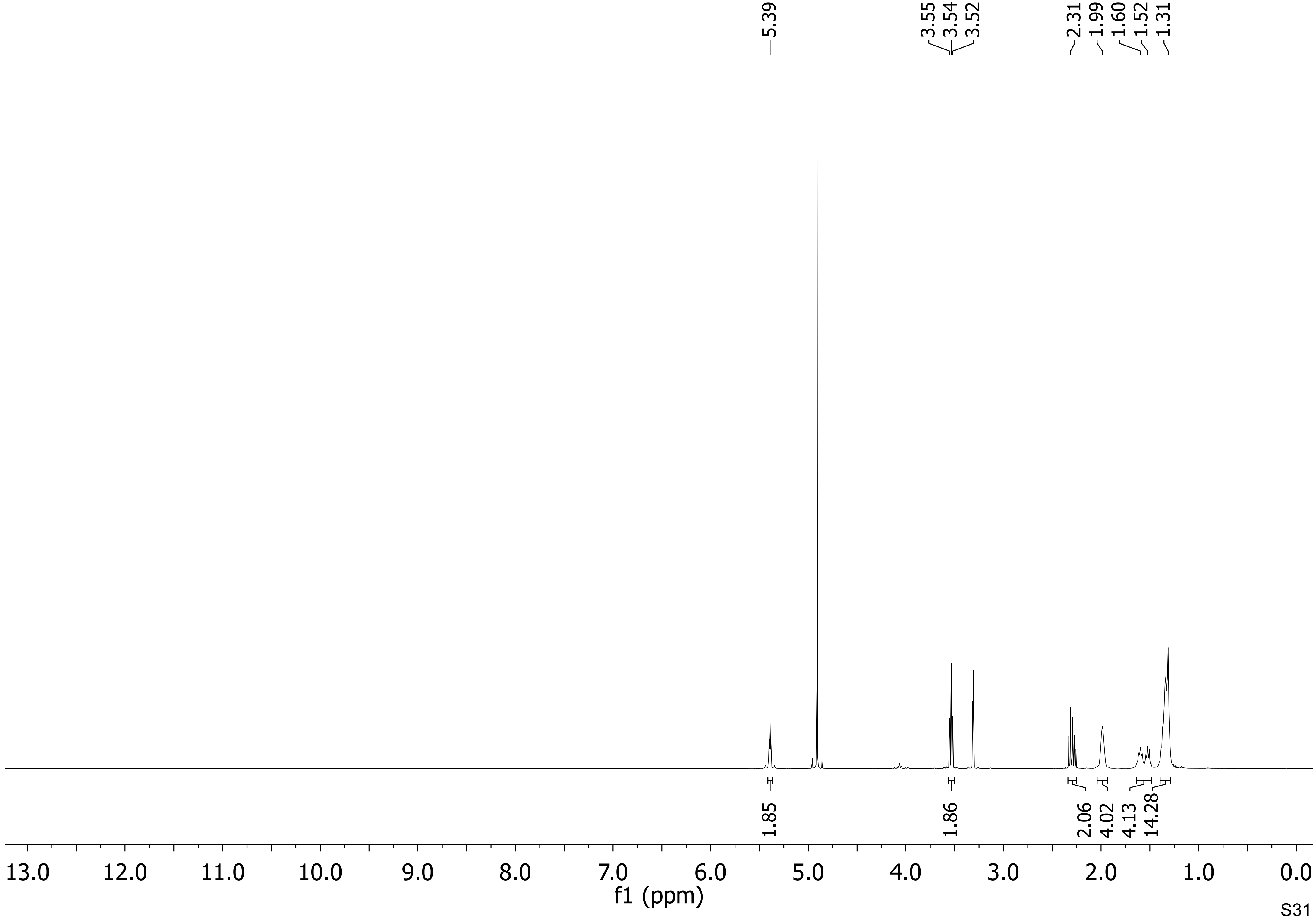

^{13}C NMR (DEPTQ, 101 MHz, CD_3OD) for compound **6a**

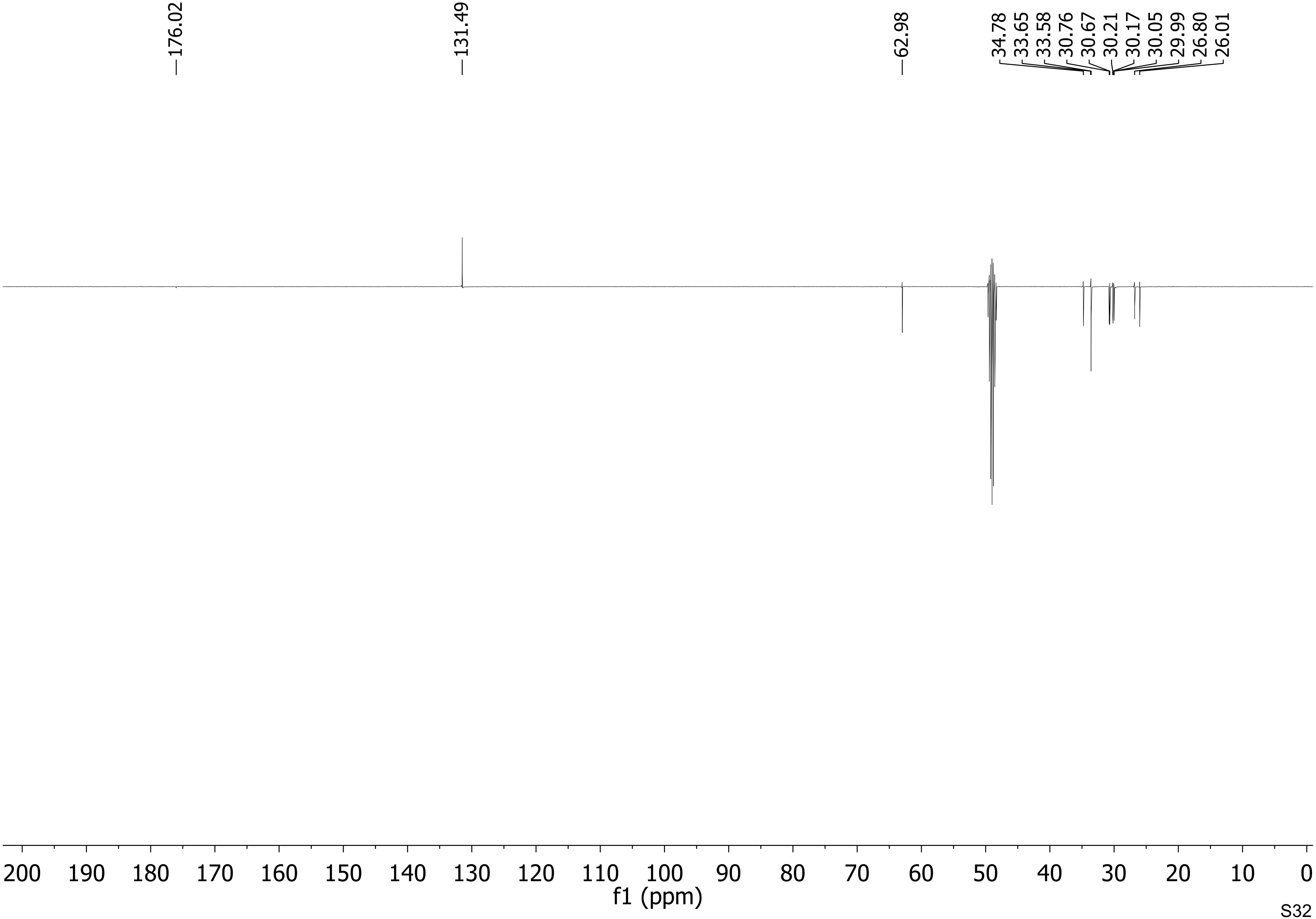

^1H NMR (400 MHz, CD_3OD) for compound **6b**

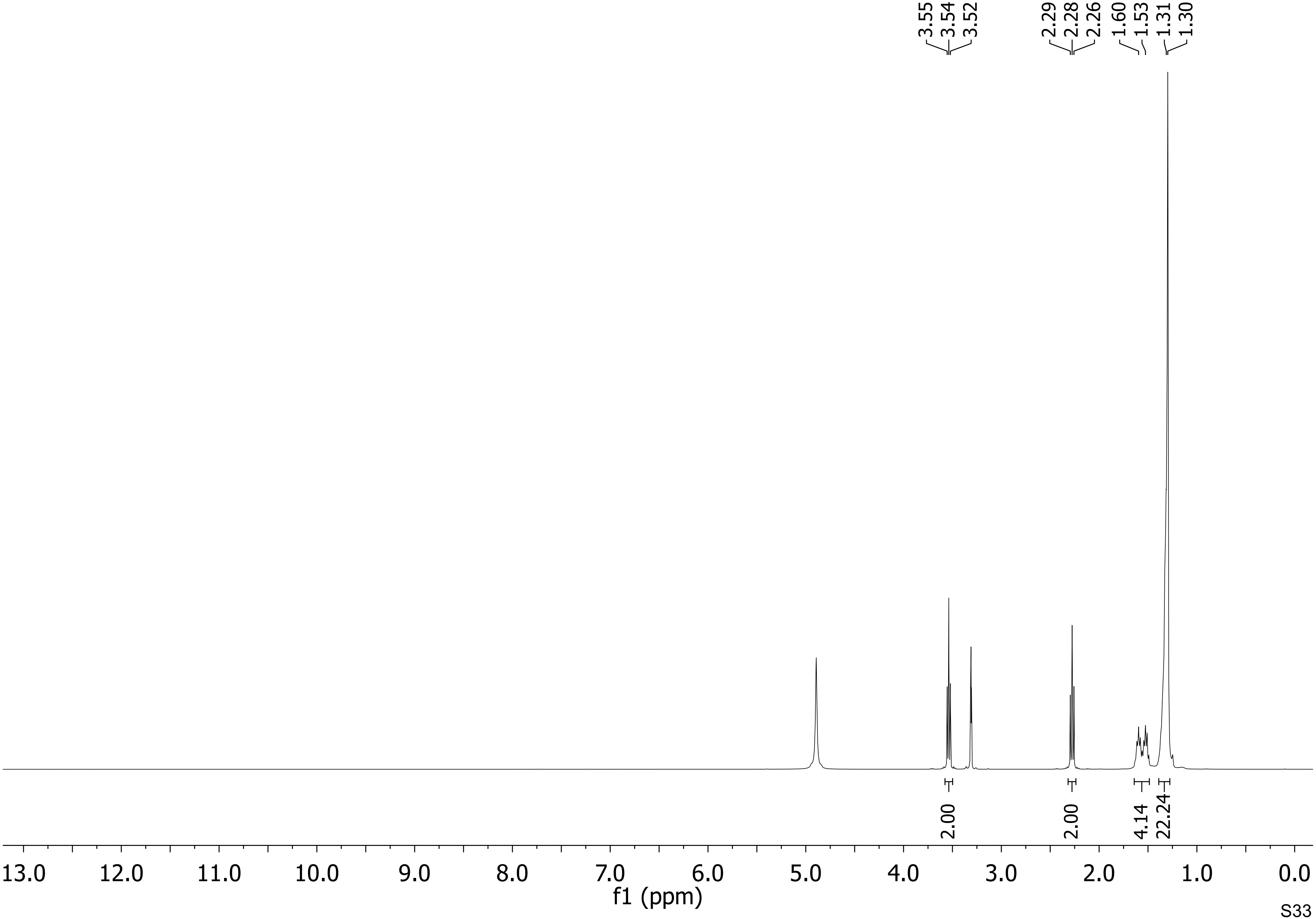

^{13}C NMR (DEPTQ, 101 MHz, CD_3OD) for compound **6b**

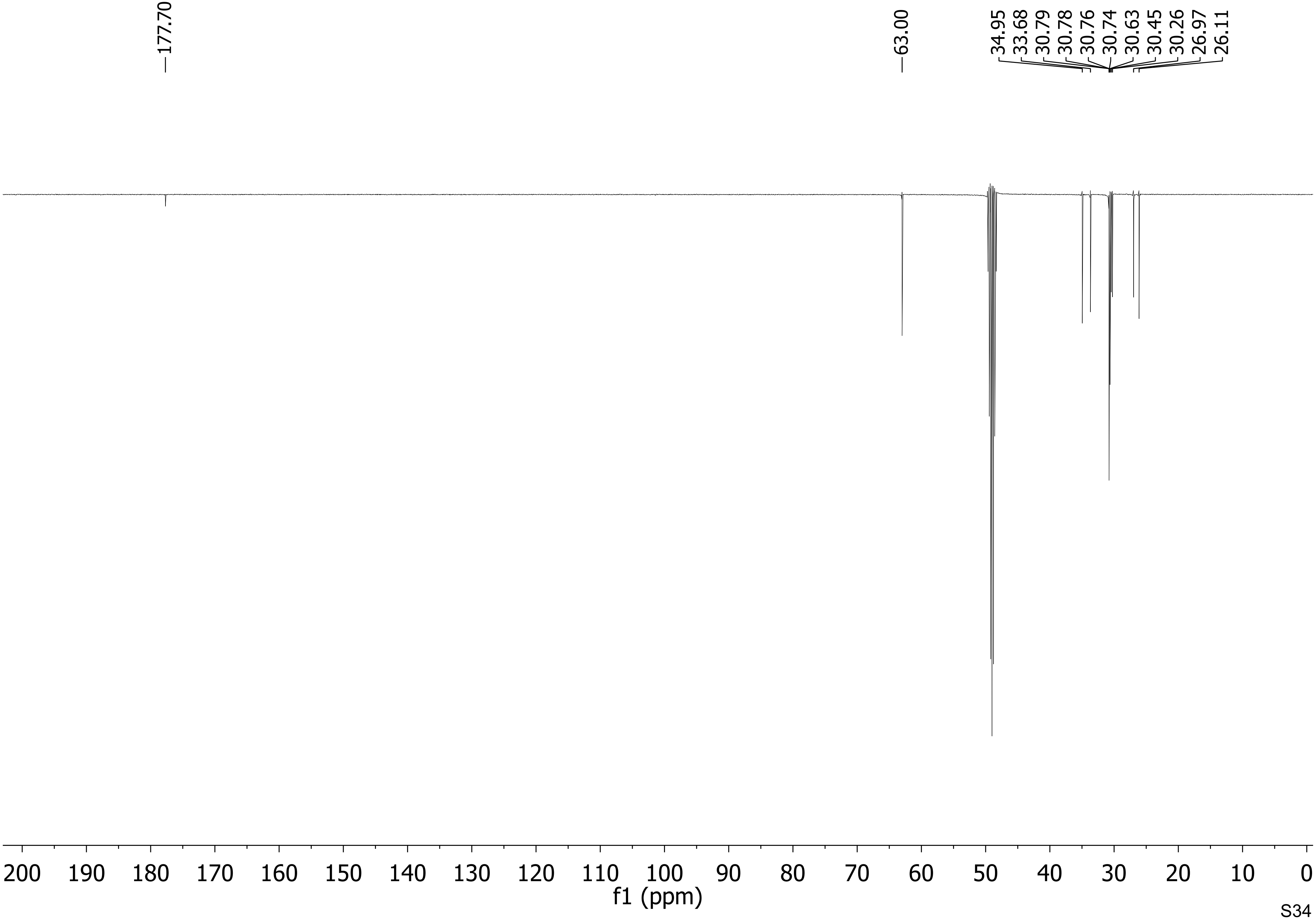

^{1}H NMR (400 MHz, $CDCl_3$) for compound **7a**

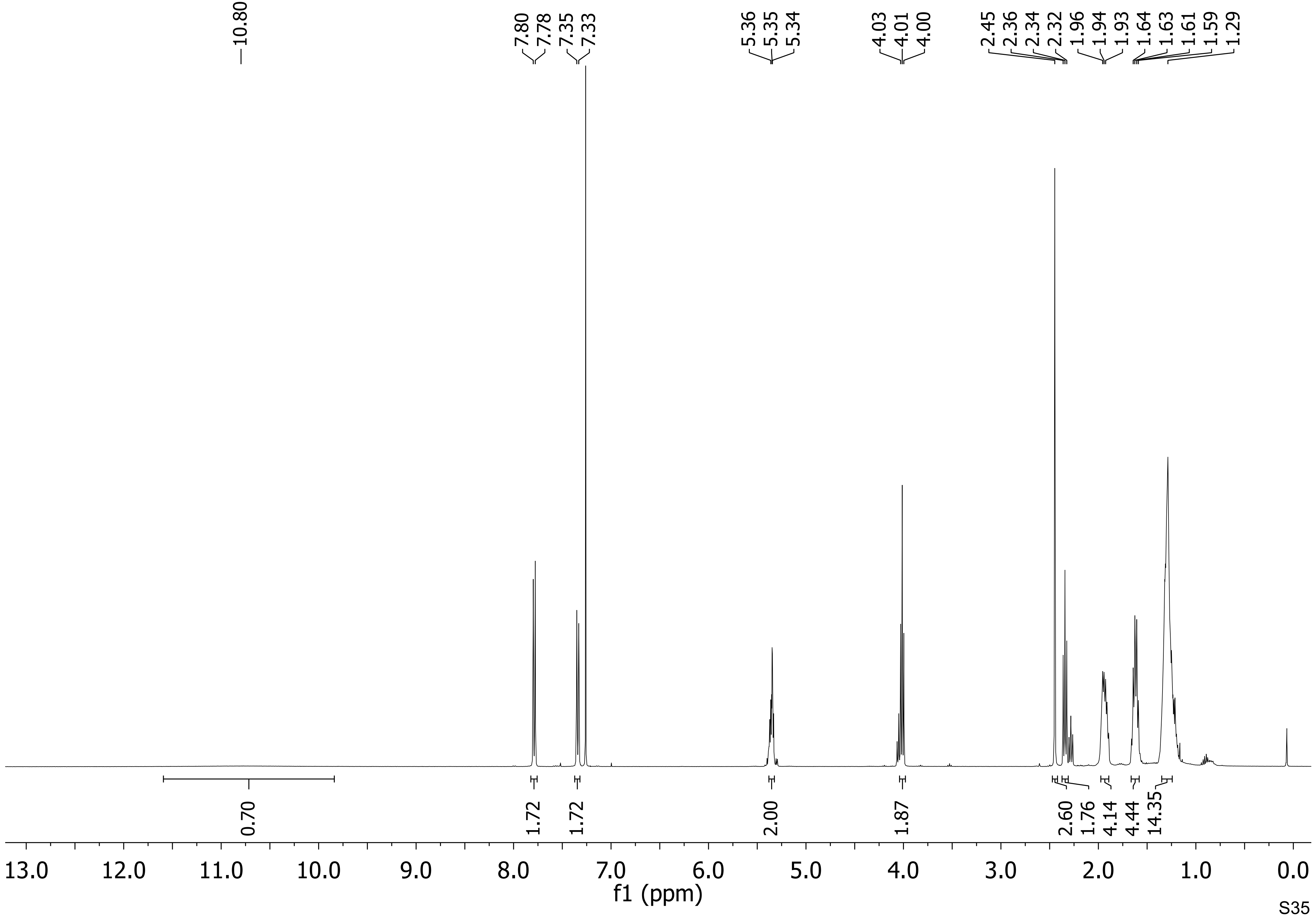

^1H NMR (400 MHz, $CDCl_3$) for compound **7a**

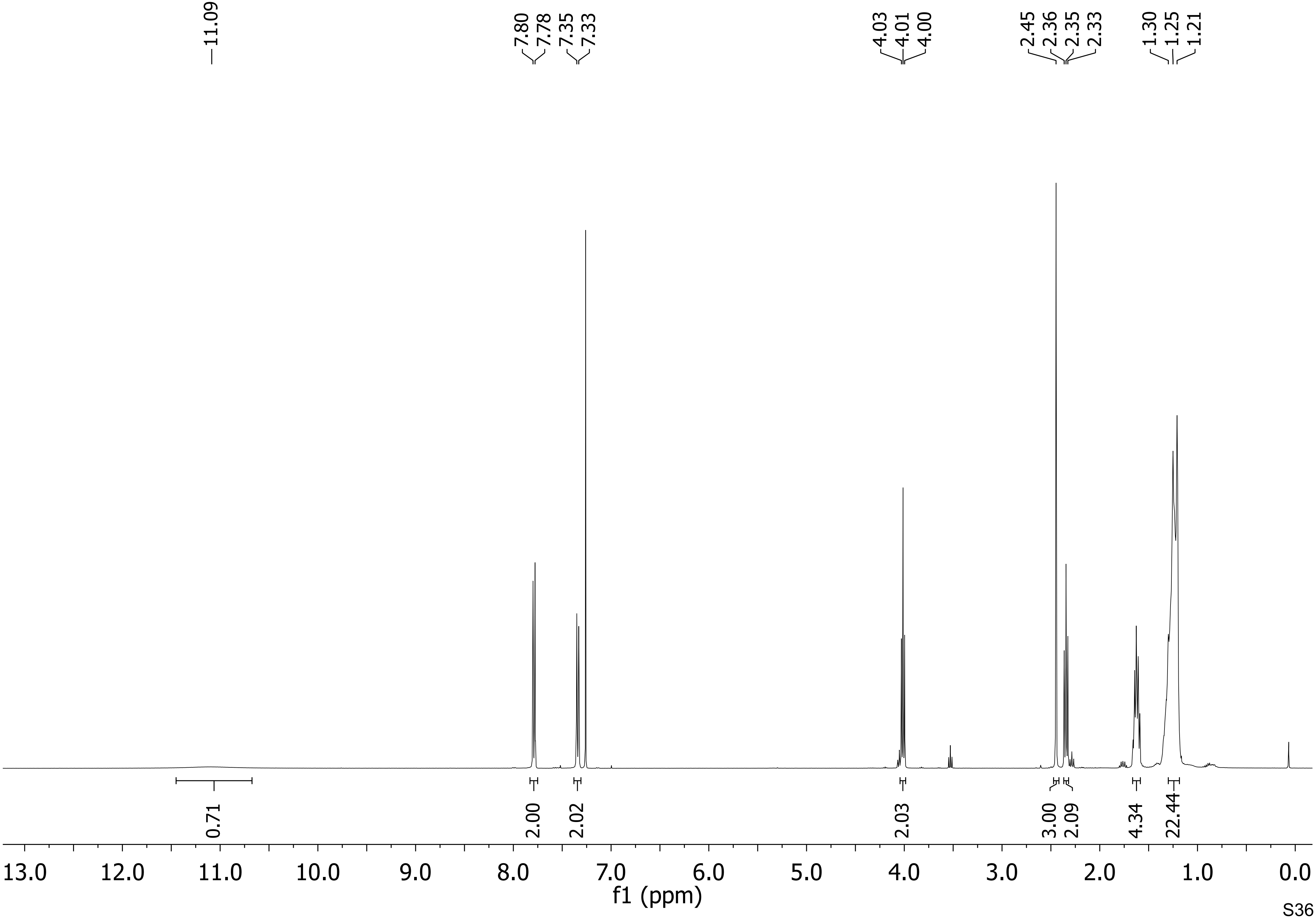

^{1}H NMR (400 MHz, $CDCl_3$) for compound **8a**

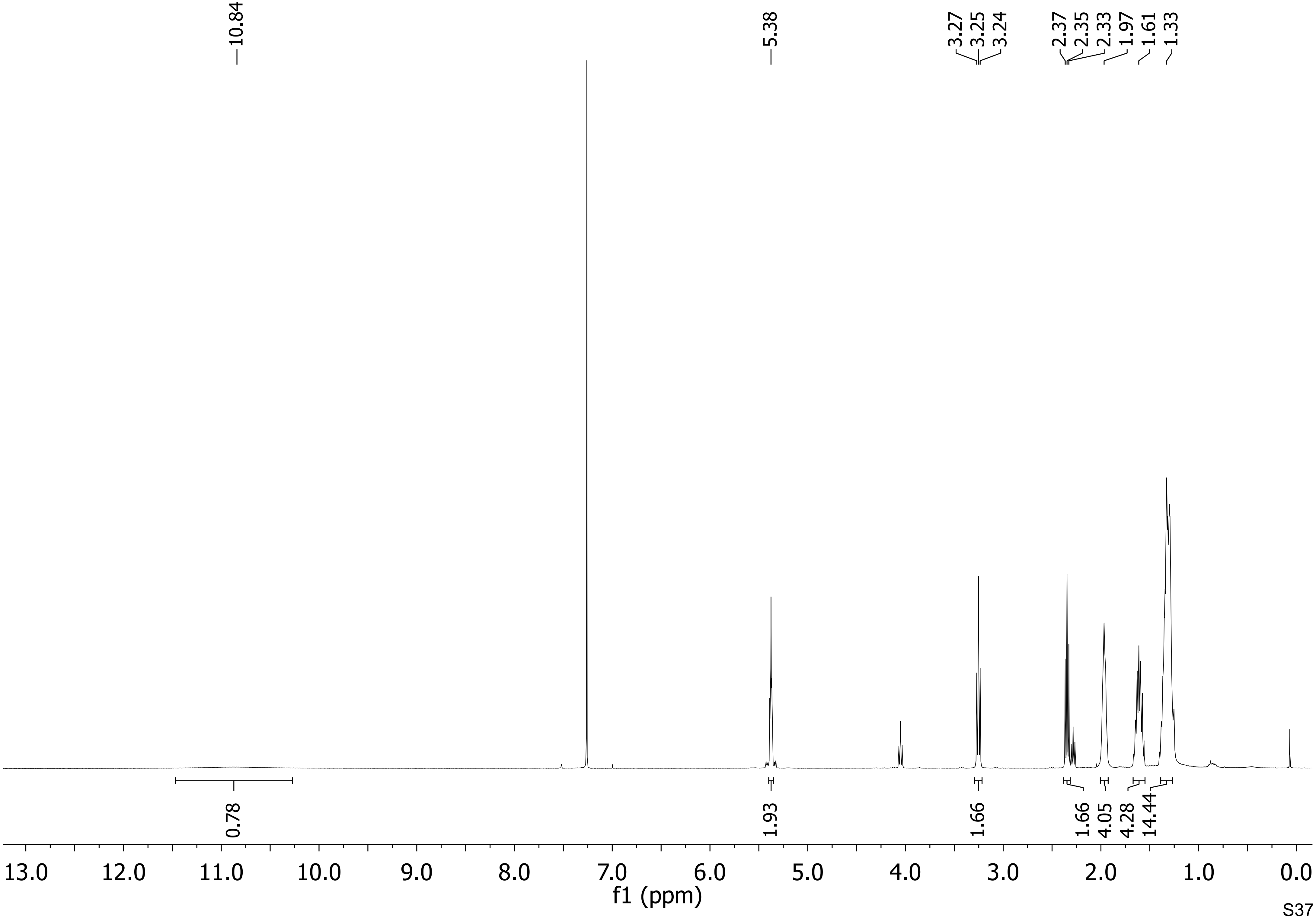

^{1}H NMR (400 MHz, $CDCl_3$) for compound **8b**

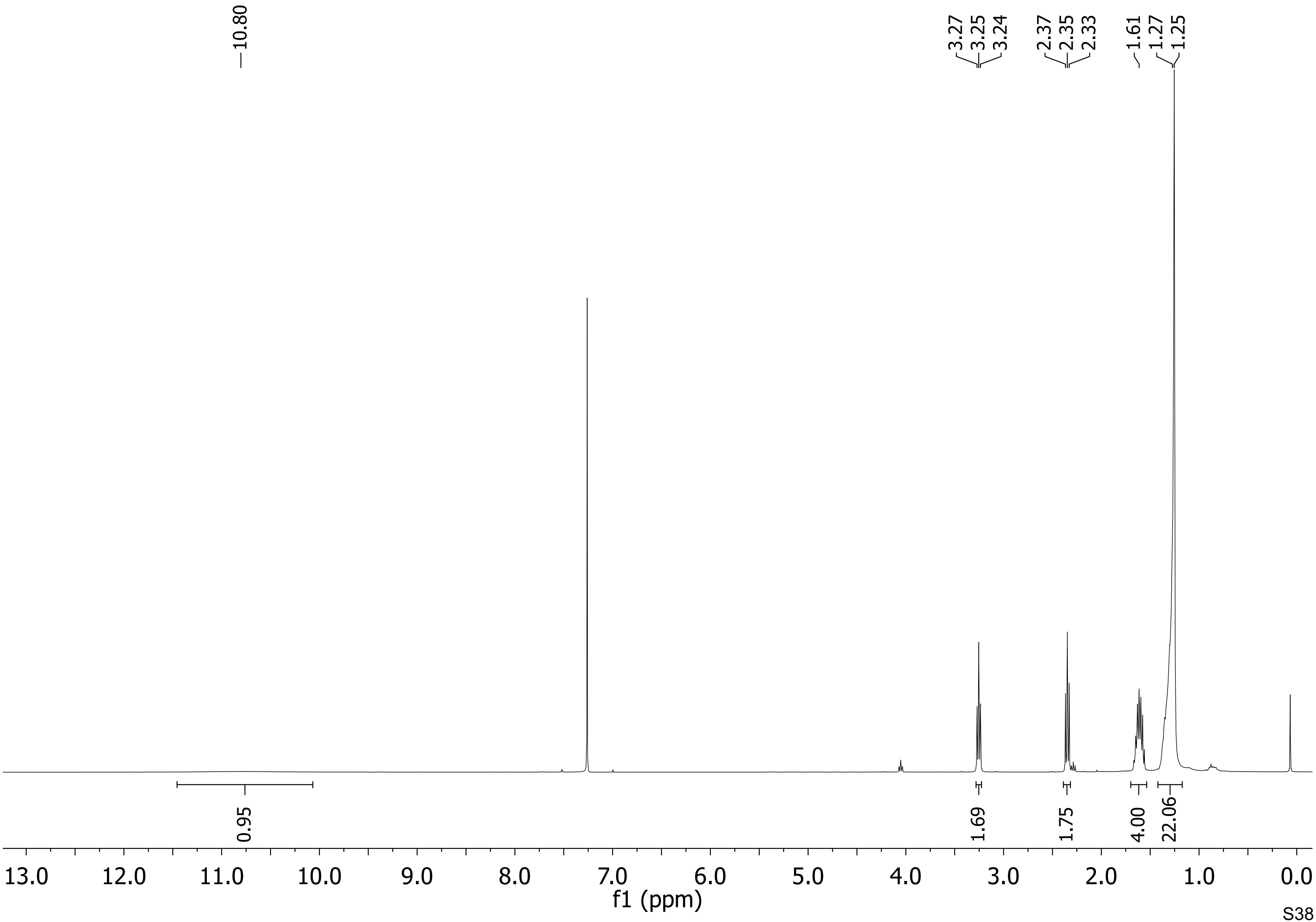

^{1}H NMR (400 MHz, $CDCl_3$) for compound **9a**

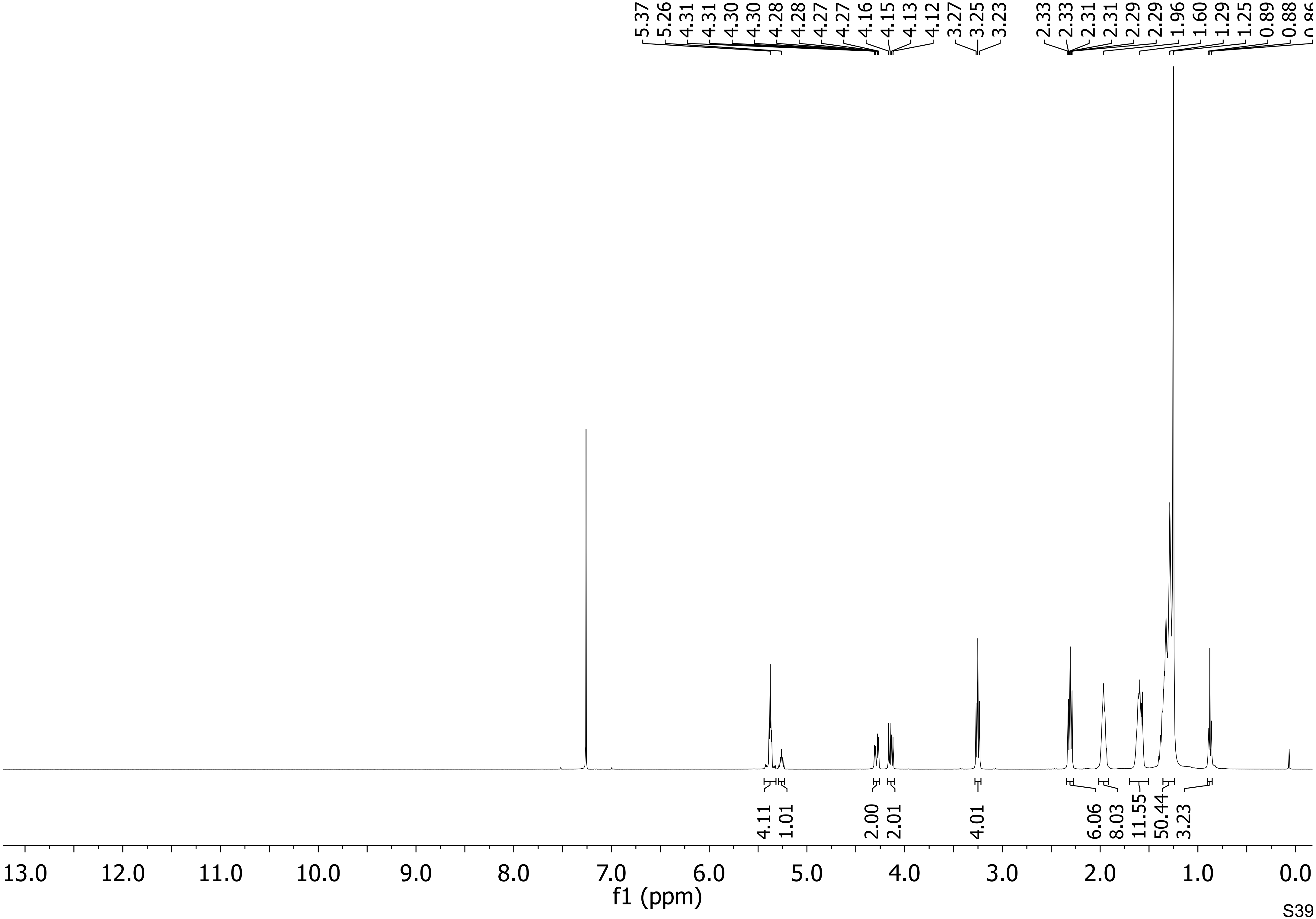

^{1}H NMR (400 MHz, $CDCl_3$) for compound **9b**

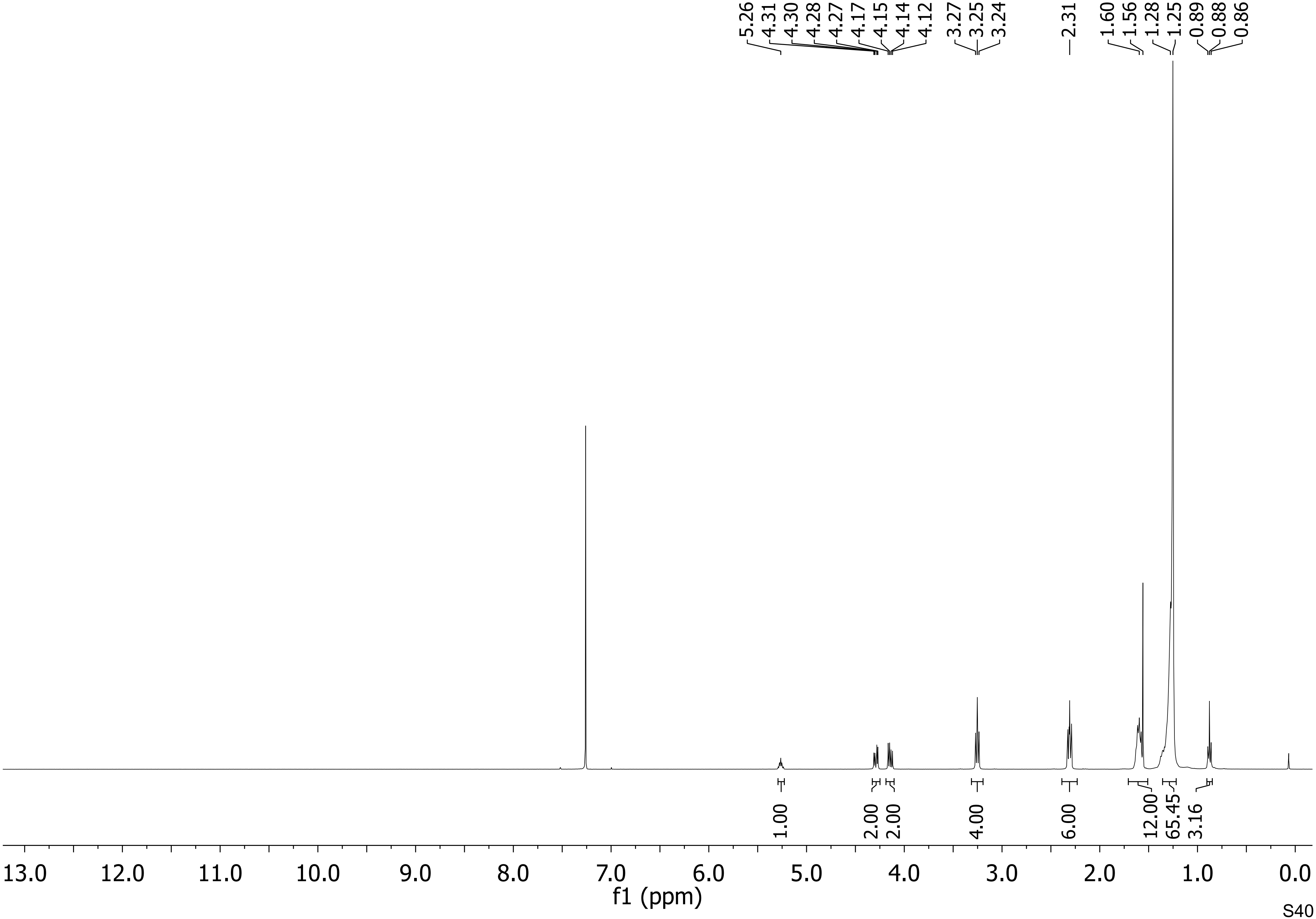

^1H NMR (400 MHz, $CDCl_3$) for glycerol trioleate oxidation (0 hours)

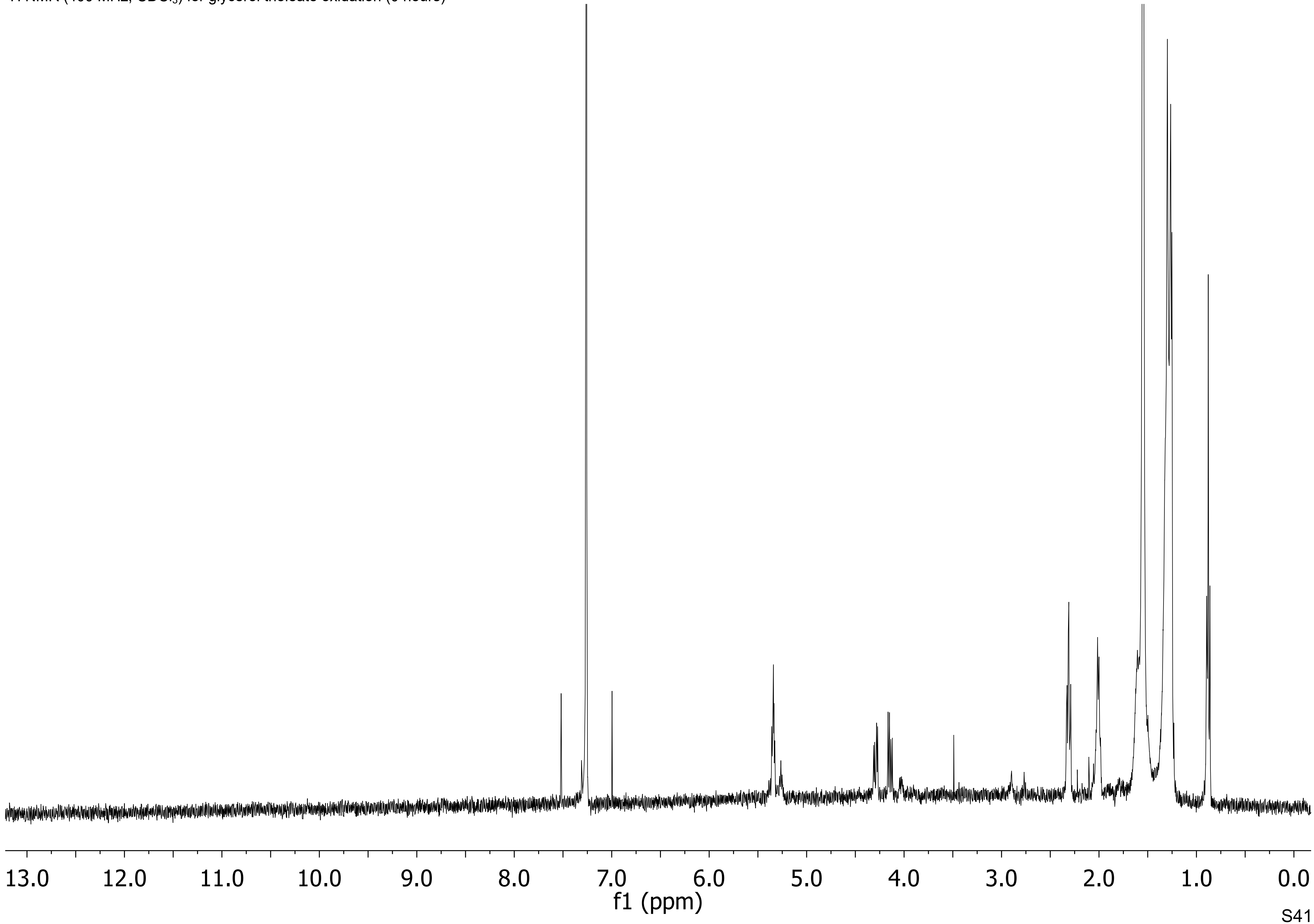

^1H NMR (400 MHz, $CDCl_3$) for glycerol trioleate oxidation (24 hours)

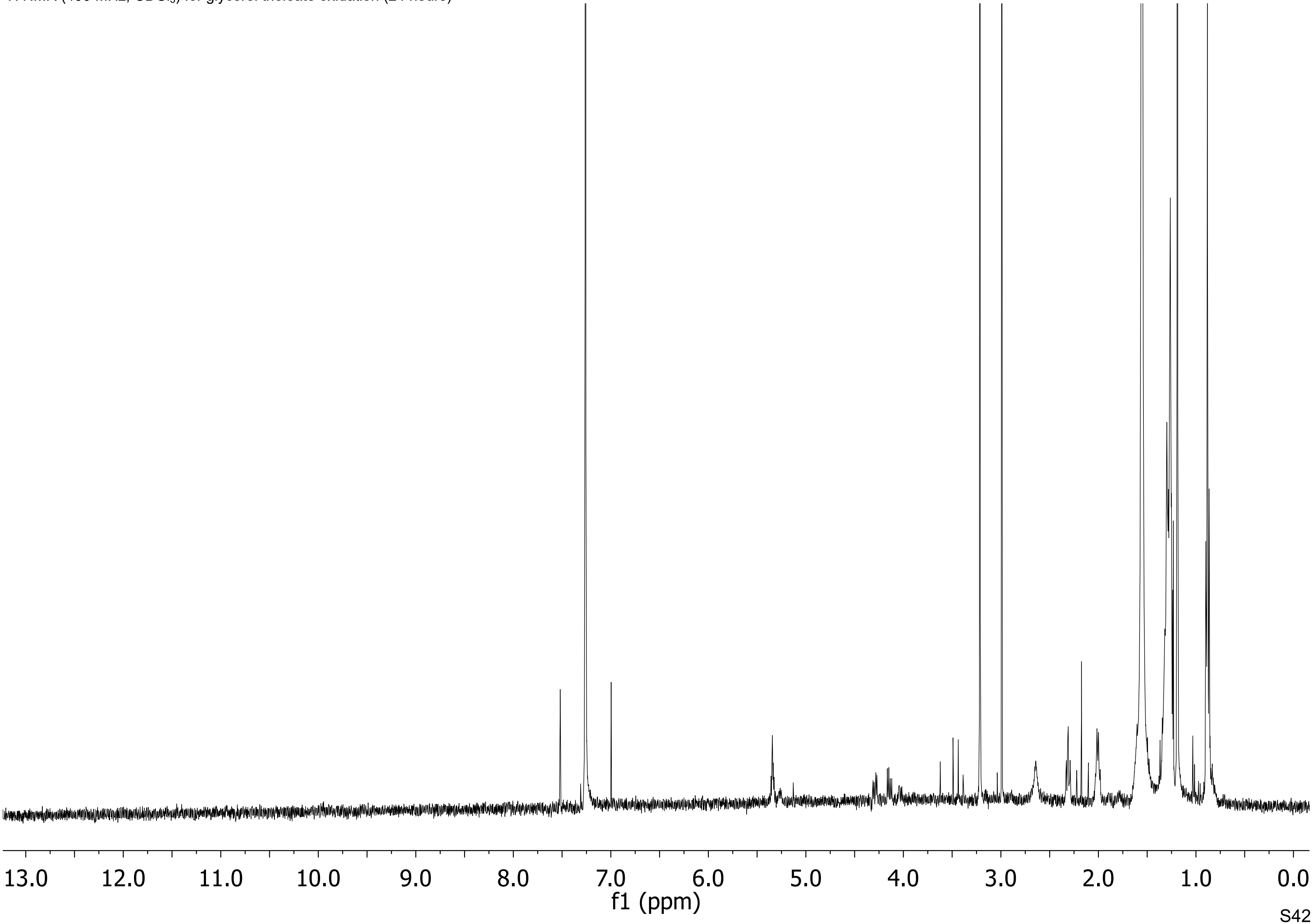

^{1}H NMR (400 MHz, $CDCl_3$) for glycerol trioleate oxidation (120 hours)

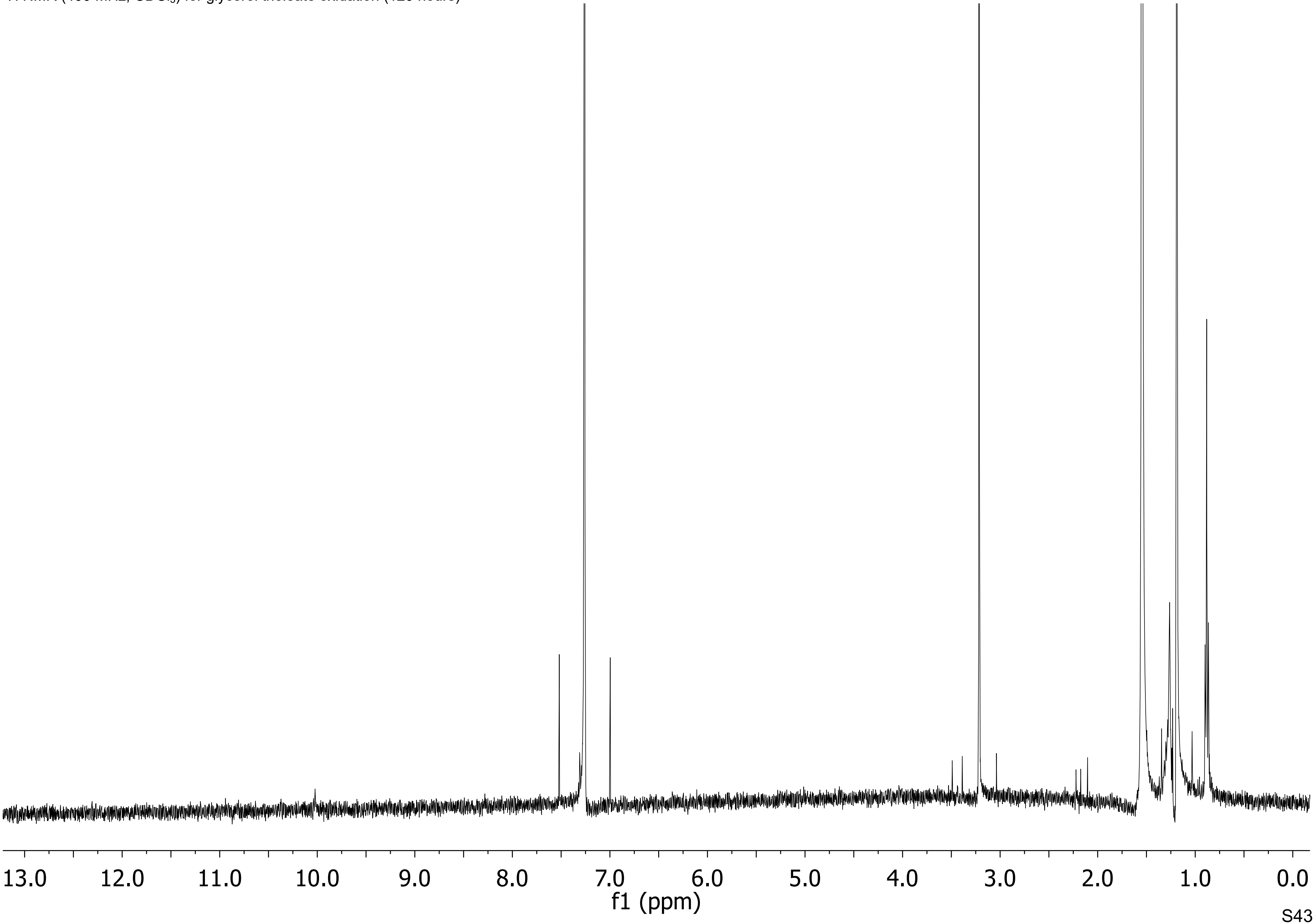

Supplementary HRMS Spectra

HRMS spectra of **1a** (MAH16):

Display Report

Analysis Info

Analysis Name	D:\Data\da74temu\Handke\MHHa-MAH16_ESIpos.d	Acquisition Date	5/8/2023 3:45:42 PM
Method	DirectInfusion_MS_pos_Na-formiate_2020_AS.m	Operator	Demo User
Sample Name	MAH 16	Instrument	timsTOF Pro 1854399.00066
Comment	50 ng/mL		

Acquisition Parameter

Source Type	ESI	Ion Polarity	Positive	Set Nebulizer	0.4 Bar
Focus	Active	Set Capillary	4500 V	Set Dry Heater	200 °C
Scan Begin	100 m/z	Set End Plate Offset	-500 V	Set Dry Gas	4.0 l/min
Scan End	1500 m/z	Set Collision Cell RF	400.0 Vpp	Set Divert Valve	Waste

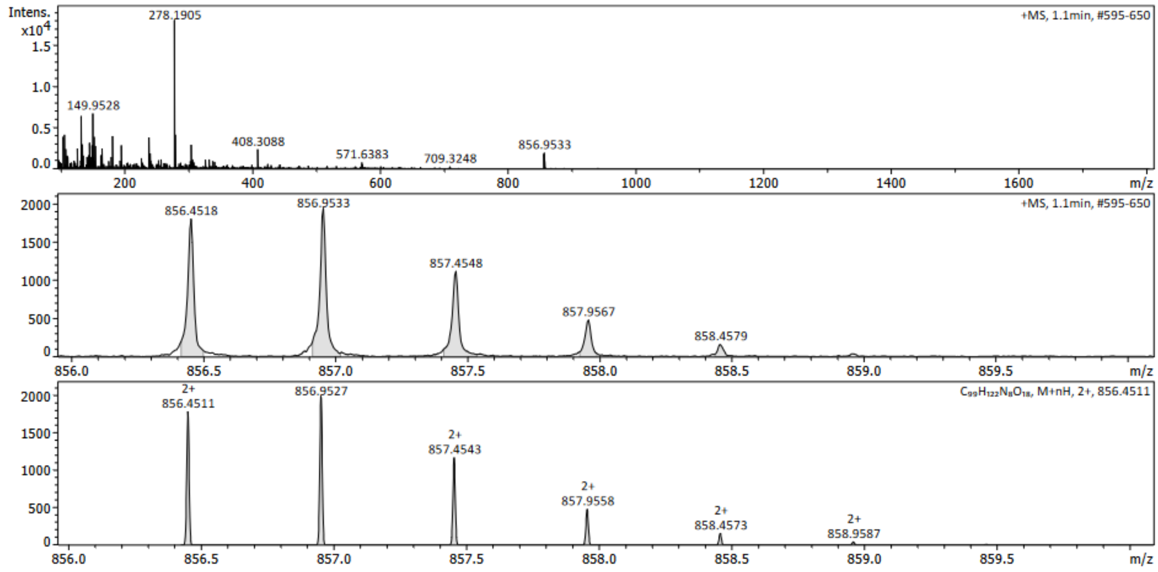

Mass Spectrum SmartFormula Report

Analysis Info

Analysis Name	D:\Data\da74temu\Handke\MHHa-MAH16_ESIpos.d	Acquisition Date	5/8/2023 3:45:42 PM
Method	DirectInfusion_MS_pos_Na-formiate_2020_AS.m	Operator	Demo User
Sample Name	MAH 16	Instrument	timsTOF Pro 1854399.00066
Comment	50 ng/mL		

Acquisition Parameter

Source Type	ESI	Ion Polarity	Positive	Set Nebulizer	0.4 Bar
Focus	Active	Set Capillary	4500 V	Set Dry Heater	200 °C
Scan Begin	100 m/z	Set End Plate Offset	-500 V	Set Dry Gas	4.0 l/min
Scan End	1500 m/z	Set Collision Cell RF	400.0 Vpp	Set Divert Valve	Waste

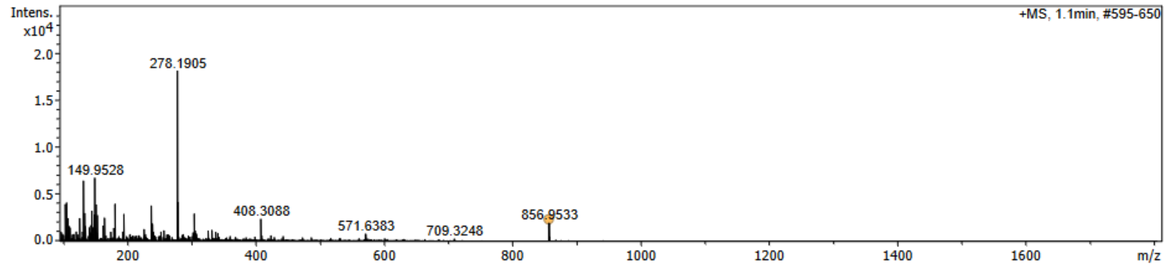

Meas. m/z	#	z	Ion Formula	m/z	err [ppm]	mSigma	#mSigma	Score	rdb	e⁻ Conf	N-Rule
856.4518	1	2+	$C_{99}H_{124}N_8O_{18}$	856.4511	-0.8	17.7	1	100.00	43.0	even	ok

HRMS spectra of **1b** (MAH12):

Display Report

Analysis Info

Analysis Name	D:\Data\da74temu\Handke\MHHa-MAH12_ESIpos.d	Acquisition Date	5/9/2023 8:22:31 AM
Method	DirectInfusion_MS_pos_Na-formiate_2020_AS.m	Operator	Demo User
Sample Name	MAH 12	Instrument	timsTOF Pro 1854399.00066
Comment	50 ng/mL		

Acquisition Parameter

Source Type	ESI	Ion Polarity	Positive	Set Nebulizer	0.4 Bar
Focus	Active	Set Capillary	4500 V	Set Dry Heater	200 °C
Scan Begin	100 m/z	Set End Plate Offset	-500 V	Set Dry Gas	4.0 l/min
Scan End	1800 m/z	Set Collision Cell RF	400.0 Vpp	Set Divert Valve	Waste

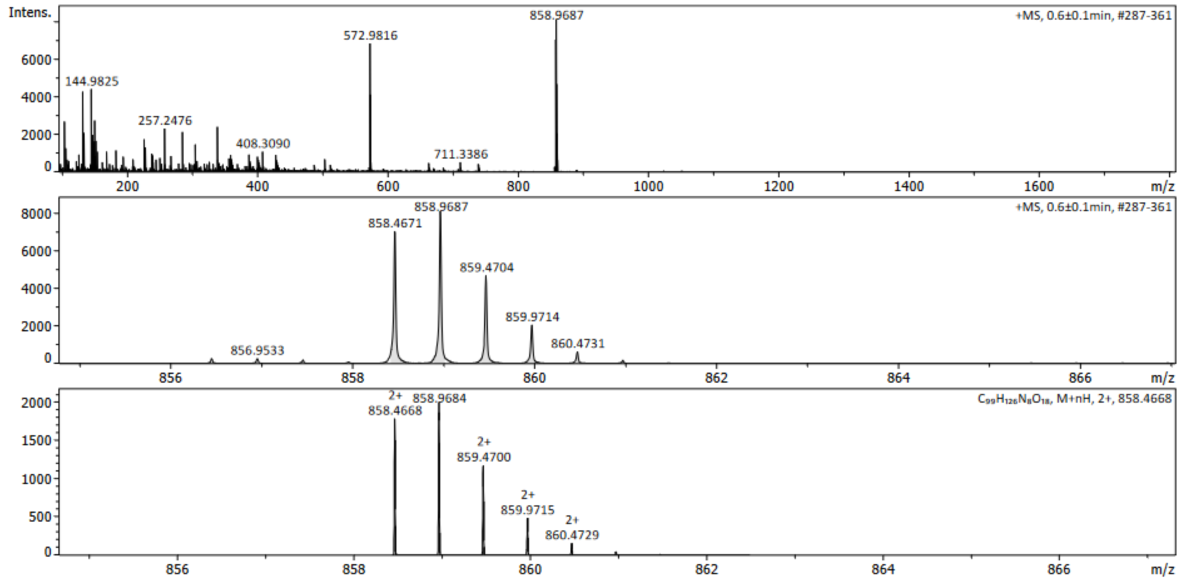

Bruker Compass DataAnalysis 6.1 printed: 5/9/2023 8:30:46 AM by: demo Page 1 of 1

Mass Spectrum SmartFormula Report

Analysis Info

Analysis Name	D:\Data\da74temu\Handke\MHHa-MAH12_ESIpos.d	Acquisition Date	5/9/2023 8:22:31 AM
Method	DirectInfusion_MS_pos_Na-formiate_2020_AS.m	Operator	Demo User
Sample Name	MAH 12	Instrument	timsTOF Pro 1854399.00066
Comment	50 ng/mL		

Acquisition Parameter

Source Type	ESI	Ion Polarity	Positive	Set Nebulizer	0.4 Bar
Focus	Active	Set Capillary	4500 V	Set Dry Heater	200 °C
Scan Begin	100 m/z	Set End Plate Offset	-500 V	Set Dry Gas	4.0 l/min
Scan End	1800 m/z	Set Collision Cell RF	400.0 Vpp	Set Divert Valve	Waste

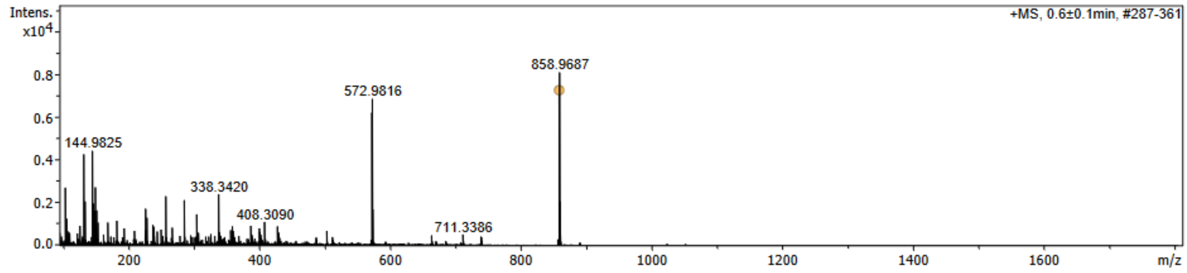

Meas. m/z	#	z	Ion Formula	m/z	err [ppm]	mSigma	#mSigma	Score	rdb	e⁻ Conf	N-Rule
858.4671	1	2+	$C_{99}H_{128}N_8O_{18}$	858.4668	-0.3	11.9	1	100.00	41.0	even	ok

Bruker Compass DataAnalysis 6.1 printed: 5/9/2023 8:31:47 AM by: demo Page 1 of 1

Supplementary HPLC Chromatograms

HPLC chromatogram of the compound **1a**:

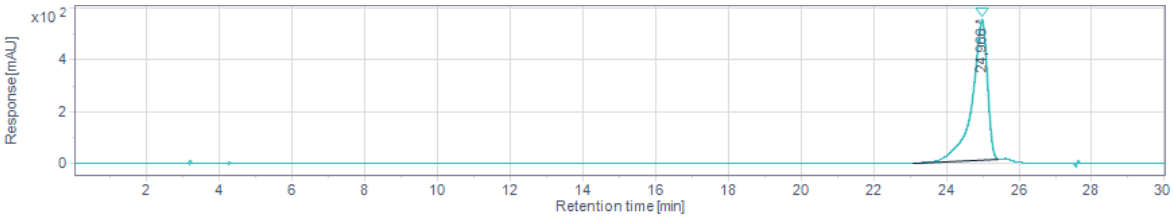

HPLC chromatogram of the compound **1b**:

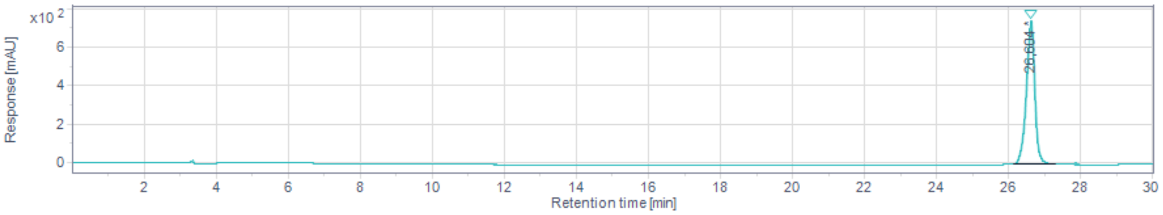

Supplementary References

1. Ni G, Li Z, Liang K, Wu T, De Libero G, Xia C. Synthesis and evaluation of immunostimulant plasmalogen lysophosphatidylethanolamine and analogues for natural killer T cells. Bioorg Med Chem. 2014;22:2966-73. https://doi.org/10.1016/j.bmc.2014.04.012.
2. Lisa M, Holcapek M. Characterization of triacylglycerol enantiomers using chiral HPLC/APCI-MS and synthesis of enantiomeric triacylglycerols. Anal Chem. 2013;85:1852-9. https://doi.org/10.1021/ac303237a.
3. Ragno D, Brandolese A, Urbani D, Di Carmine G, De Risi C, Bortolini O, Giovannini PP, Massi A. Esterification of glycerol and solketal by oxidative NHC-catalysis under heterogeneous batch and flow conditions. React Chem Eng. 2018;3:816-25. https://doi.org/10.1039/C8RE00143J.
4. Kurzhals S, Zirbs R, Reimhult E. Synthesis and magneto-thermal actuation of iron oxide core-PNIPAM shell nanoparticles. ACS Appl Mater Interfaces. 2015;7:19342-52. https://doi.org/10.1021/acsami.5b05459.
5. Dougan H, Lyster DM, Vincent JS. Macrocyclic lactones as a source for radiohalogenated fatty acid analogs and their precursors. J Radioanal Nucl Chem 1985;89:71-8. https://doi.org/https://doi.org:10.1007/BF02070205
6. Pattipeiluhu R, Crielaard S, Klein-Schiphorst I, Florea BI, Kros A, Campbell F. Unbiased identification of the liposome protein corona using photoaffinity-based chemoproteomics. ACS Cent Sci. 2020;6:535-45. https://doi.org/10.1021/acscentsci.9b01222.
7. Brun MA, Tan KT, Griss R, Kielkowska A, Reymond L, Johnsson K. A semisynthetic fluorescent sensor protein for glutamate. J Am Chem Soc. 2012;134:7676-8. https://doi.org/10.1021/ja3002277.
